# Integrating Transcriptome and Coexpression Network Analyses to Characterize Salicylic Acid- and Jasmonic Acid-Related Genes in Tolerant Poplars Infected with Rust

**DOI:** 10.3390/ijms22095001

**Published:** 2021-05-08

**Authors:** Qiaoli Chen, Ruizhi Zhang, Danlei Li, Feng Wang

**Affiliations:** 1 Key Laboratory of Alien Forest Pests Detection and Control-Heilongjiang Province, School of Forestry, Northeast Forestry University, Harbin 150040, China; qiaolichen@nefu.edu.cn (Q.C.); zhangruizhi@nefu.edu.cn (R.Z.); danleili@nefu.edu.cn (D.L.); 2 Key Laboratory of Sustainable Forest Ecosystem Management-Ministry of Education, Northeast Forestry University, Harbin 150040, China

**Keywords:** *Melampsora larici-populina*, *Populus*, jasmonic acid, salicylic acid, WGCNA

## Abstract

*Melampsora larici-populina* causes serious poplar foliar diseases called rust worldwide. Salicylic acid (SA) and jasmonic acid (JA) are important phytohormones that are related to plant defence responses. To investigate the transcriptome profiles of SA- and JA-related genes involved in poplar rust interaction, two tolerant poplars and one intolerant poplar were selected for this study. Weighted gene coexpression network analysis (WGCNA) was applied to characterize the changes in the transcriptome profiles and contents of SA and JA after infection with the virulent E4 race of *M. larici-populina*. In response to infection with the E4 race of *M. larici-populina*, tolerant symptoms were correlated with the expression of genes related to SA and JA biosynthesis, the levels of SA and JA, and the expression of defence-related genes downstream of SA and JA. Tolerant poplars could promptly regulate the occurrence of defence responses by activating or inhibiting SA or JA pathways in a timely manner, including regulating the expression of genes related to programmed cell death, such as *Kunitz-type trypsin inhibitor* (*KTI*), to limit the growth of E4 and protect themselves. WGCNA suggested that *KTI* might be regulated by a *Cytochrome P450 family* (*CYP*) gene. Some *CYP*s should play an important role in both JA- and SA-related pathways. In contrast, in intolerant poplar, the inhibition of SA-related defence signalling through increasing JA levels in the early stage led to continued inhibition of a large number of plant–pathogen interaction-related and signalling-related genes, including *NBS-LRR*s, *EDS1*, *NDR1*, *WRKY*s, and *PR*s. Therefore, timely activation or inhibition of the SA or JA pathways is the key difference between tolerant and intolerant poplars.

## 1. Introduction

Rust caused by *Melampsora* spp. is one of the most damaging and widely distributed diseases of poplar leaves, and the most widespread and frequent causative agent is *M. larici-populina* [1]. In Europe in the mid-20th century, hybrids between *Populus deltoides*, *P. nigra* or *P. trichocarpa* were selected for their immunity to rust [2,3]. However, breakdown of resistance to *M. larici-populina* in clones of these hybrids began to be detected in the early 1980s with the appearance of new races of *M. larici-populina* [4,5,6], and outbreaks of rust on these hybrids were caused by a new race, E4 [7]. It is often difficult to find an E4-resistant poplar since many poplars with excellent cultivation characteristics are susceptible. However, the results of inoculation tests showed that some hybrid poplars were tolerant to infection by specific rust races. For example, E4 can only generate a few urediniospores over a life cycle on the leaves of *P. deltoides* × *P. trichocarpa* and *P. trichocarpa* × *P. deltoides*. In addition, the survival time of infected leaves on branches was much longer than that of the intolerant poplar *P. nigra* × *P. deltoides*. This interesting phenomenon suggested that these tolerant poplars were in the intermediate state of E4 susceptibility and E4 resistance. The most obvious difference is that tolerant poplars could develop a hypersensitive cell death response (HR) at the infection site. The initial manifestation of plant infection with pathogens is the HR, which prompts the rapid death of infected plant cells, thereby preventing the spread of pathogens from the invasion site to adjacent healthy tissues. Further study of the molecular mechanism of tolerance may provide new insight into how rust overcomes poplar resistance.

In the course of long-term evolution, plants have formed a complex resistance mechanism that can defend against pathogen infection [8]. The complex immune system is inducible upon plant receptor-mediated recognition of a great diversity of pathogen-derived molecules [9], mainly including the recognition of conserved pathogen-(or microbial) associated molecular patterns (P/MAMPs) by pattern recognition receptors (PRRs) [10,11], and the recognition of highly variable effectors by highly polymorphic resistance (R) proteins [8,12,13]. There is a wide range of signal molecule exchange and recognition interactions between plants and pathogens [9,13]. Small molecule compounds affect signal regulation in plants and their pathogens, which determines the outcome of the competition between them to a large extent. Phytohormones and their signal transduction networks play an important role [14,15]. In response to pathogen challenge, plant cells undergo dramatic transcriptional reprogramming. Different phytohormone signalling pathways perform different functions in the interaction between plants and pathogens [14,16,17,18,19,20,21], and the pathways are interrelated and affect each other to coordinate the regulation of plant growth and development as well as external defence response, so that plants are always in a physiological balance to protect themselves to the greatest extent [14]. However, when pathogens invade plants, they can interfere with the synthesis, metabolism, and signalling molecule network of phytohormones by secreting a variety of effector proteins or directly secreting hormones or hormone analogues to plant cells to weaken the disease-resistant defence response of plants and achieve pathogenesis [13].

Salicylic acid (SA) and jasmonic acid (JA) are important natural phytohormones that promote systemic immunity in plant defence responses [16,22,23,24]. SA was proposed as an endogenous signal for plant disease resistance, as the accumulation of SA in the plant has been shown to induce the expression of pathogenesis-related proteins (PRs), which increase the resistance of plants to pathogen infection [25,26,27,28,29]. Once synthesized, SA may undergo a number of biologically relevant chemical modifications to produce inactive forms [30,31,32,33,34,35,36,37]; however, the active molecule in planta is believed to be free SA [38]. These inactive molecules can be stored until required to activate SA-triggered responses [39]. Although there are exceptions, SA-dependent defences are generally effective against biotrophic pathogens, JA-dependent defences are generally effective against necrotrophic pathogens, and there is cross-talk and antagonism between the SA and JA signalling pathways [40,41]. Cross-talk between the SA and JA signalling pathways has become an important mechanism for plants to control their induced defence responses and potentially reduce the cost of defence-related adaptation [40,42,43]. Transcriptome analyses revealed extensive interactions between the two pathways; among them, the antagonistic effects of SA on JA-responsive gene expression were the most prominent [43,44,45]. In *Arabidopsis thaliana*, activation of the SA pathway inhibits a large number of JA-responsive genes, including the JA marker genes *PLANT DEFENSIN1.2* (*PDF1.2*) and *VEGETATIVE STORAGE PROTEIN2* (*VSP2*) [46,47]. Other hormones, such as ethylene, abscisic acid, gibberellins, auxins, and cytokinins, also affect plant immunity, often via the modulation of the SA and JA signalling pathways [40,48,49]. These pathways influence each other through a complex network of regulatory interactions rather than functioning independently [50]. The contribution of these signalling molecules to plant defence differs depending on the invading pathogen [20].

In this study, the changes in the transcriptome profiles and contents of SA and JA after infection with E4 in two tolerant poplars, *P. trichocarpa* × *P. deltoides* (‘Tolerant 1’) and *P. deltoides* × *P. trichocarpa* (‘Tolerant 2’), and an intolerant poplar, *P. nigra* × *P. deltoides* (‘Intolerant’), were investigated to study the different defence responses related to SA and JA in the tolerant and intolerant poplars upon the E4 infection. Weighted correlation network analysis (WGCNA) [51] was performed to obtain a better understanding of SA- and JA-related gene expression patterns in E4-infected poplars to provide markers that can be associated with changes in SA and JA.

## 2. Results

### 2.1. Disease Assessment

The maximum slope factor (MSF) of ‘Tolerant 1’ was 0.707, the MSF of ‘Tolerant 2’ was 0.774, and the MSF of ‘Intolerant’ was 1.378. The average maximum slope factor (AMSF) was 0.953. Disease scores were given as scale 0 (slope factor, SF = 0), scale 1 (0 < SF ≤ AMSF × 2/7), scale 2 (AMSF × 2/7 < SF ≤ AMSF × 4/7), scale 3 (AMSF × 4/7 < SF ≤ AMSF × 6/7), or scale 4 (SF > AMSF × 6/7). According to the SF value, the disease scores of ‘Tolerant 1’, ‘Tolerant 2’, and ‘Intolerant’ were scale 2 (SF = 0.532), scale 3 (SF = 0.575), and scale 4 (SF = 1.328), respectively. Therefore, the ranking of anti-E4 infection ability was as follows: ‘Tolerant 1’ > ‘Tolerant 2’ > ‘Intolerant’.

**Figure 1:**
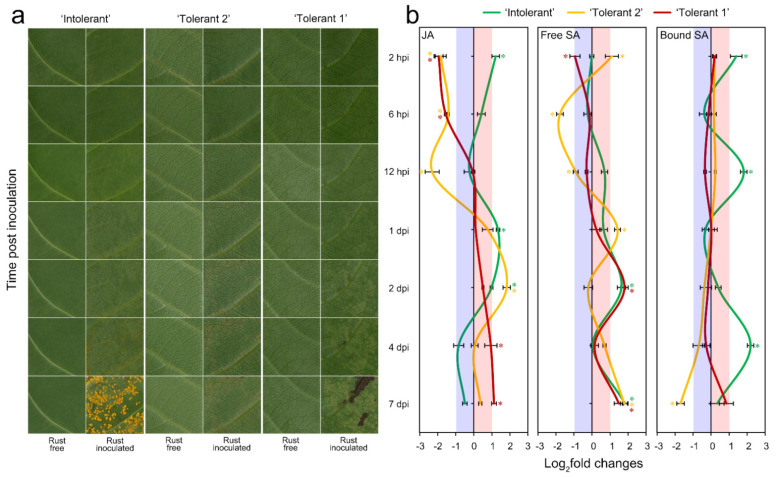
Symptoms and changes in JA and SA contents in poplar leaves infected with E4. (**a**) Symptoms of poplar leaves infected with E4. (**b**) Changes in JA and SA contents in poplar leaves infected with E4. * *p*-value < 0.01, rust inoculated leaves versus rust free leaves, *n* = 3; Hpi, hours post inoculation; dpi, days post inoculation.

E4 spores germinated at 2 h post inoculation (hpi) on leaves of all three poplars, but the growth of E4 on the tolerant poplars was significantly slower than that on ‘Intolerant’. E4 could complete a vegetative cycle in seven days on ‘Intolerant’, indicating its characteristic of E4 intolerance. Only a few new urediniospores were generated at seven 7 days post inoculation (dpi), and visible scattered lesions appeared on ‘Tolerant 2’. Barely mature urediniospores could be found at 7 dpi, and confluent necrosis appeared on ‘Tolerant 1’. These results indicated that the growth of E4 was inhibited in tolerant poplars, indicating their characteristic of E4 tolerance (Figure 1a). Although both tolerant poplars were determined to be E4 growth-inhibiting and reproduction-delaying poplars, this interpretation was based on multiple necrotic spots appearing at 7 dpi, which was also the most typical characteristic of ‘Tolerant 1’. However, ‘Intolerant’, which was susceptible, could provide enough sources for E4.

### 2.2. JA and SA Content Determination

The most significant difference was that the content of JA decreased in tolerant poplars but increased in the intolerant poplar at the beginning of E4 infection, indicating that the decrease in JA content in the early stage of infection should be beneficial to the occurrence of tolerance reactions (Figure 1b). On the other hand, the changing trends of ‘Tolerant 2’ showed a relatively larger fluctuation. The difference between ‘Tolerant 2’ and ‘Tolerant 1’ indicated that they might adopt different strategies in terms of free SA-related tolerance. In addition, the content of bound SA increased frequently (2 hpi, 12 hpi, and 4 dpi) in response to E4 infection in ‘Intolerant’, suggesting that the conversion of bound SA was more intense in the intolerant poplar.

### 2.3. Digital Gene Expression Sequencing

According to the finding that the contents of JA and SA in the three poplars were obviously different during the process of E4 infection, we hypothesized that the expression of genes related to the signalling pathways of these two phytohormones should be different. The differences in the expression of these genes might eventually lead to variations in the defence response in the three poplars. To characterize the gene transcript patterns, the inoculated leaves of the three different poplars at seven different time points (2 hpi, 6 hpi, 12 hpi, 1 dpi, 2 dpi, 4 dpi, and 7 dpi) and their corresponding control samples were selected according to the infection characteristics of E4. In total, 42 libraries were constructed and sequenced. An average of 24.50 Mb raw reads were obtained for each sample. After removing the low-quality reads, an average of 23.3 million clean reads for each sample were obtained (Table S1). The dataset was deposited in the Sequence Read Archive (SRA, accession No. SRR4302070). A total of 37,454 assembled unigenes were generated from the 42 libraries.

### 2.4. WGCNA Revealed Modules Highly Related to JA and SA

After obtaining the gene expression patterns of the three different poplars at different time points after E4 infection, WGCNA was applied to obtain the gene sets with strong correlations with JA or SA. To eliminate noise from genes that were not expressed, we filtered probes with median FPKM (fragments per kilobase per million mapped fragments) levels that did not exceed 1. The expression values of 24,054 genes in 42 transcriptomes were obtained and used to construct the coexpression module with WGCNA package tools. Cluster analysis was performed on these samples to determine that there were no obvious outliers (Figure S1). We chose a power of 12, which was the lowest power at which the scale-free topology fit index curve flattened out upon reaching a high value (in this case, approximately 0.87, Figure S2), to construct coexpression modules. Thirty-eight distinct gene coexpression modules were identified (Figure S3) to separate out genes with similar expression patterns. These coexpression modules were constructed and shown in different colours, and the number of genes in each module is shown in Table S2 (grey was reserved for unassigned genes). Interactions of the 38 coexpression modules were analysed, and the gene network was visualized by plotting a heatmap (Figure S4). Each set of highly correlated genes corresponded to a branch of the tree. There was often a high topological overlap between genes in the same module.

**Figure 2:**
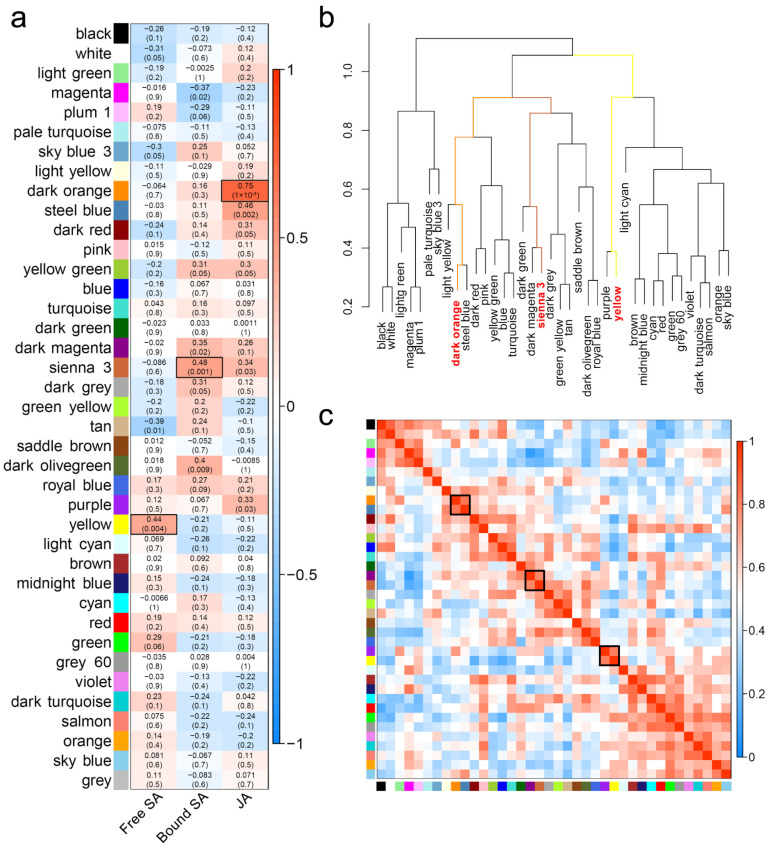
WGCNA revealed the modules highly related to JA or SA. (**a**) Module-trait associations. Each row corresponds to an ME, and each column corresponds to a trait. Each cell contains the corresponding correlation and *p*-value. The table is colour-coded by correlation according to the colour legend. (**b**) The hierarchical clustering dendrogram of correlated MEs. (**c**) The heatmap of correlated MEs.

The module eigengene (ME) is defined as the first principal component of a given module. It can be considered representative of the gene expression profiles in a module. Modules with common expression pattern interactions in the coexpression modules that were associated with particular traits were identified based on the correlation between the ME and the trait (Figure 2a). The analysis revealed that the dark orange module was significantly associated with JA (correlation value, cor = 0.75, *p*-value = 1 × 10^−8^), the ‘sienna 3’ module was highly associated with bound SA (cor = 0.48, *p*-value = 0.001), and the yellow module was highly associated with free SA (cor = 0.44, *p*-value = 0.004).

Groups of correlated MEs were identified (Figure 2b,c). The dark orange module and the steel blue module were highly related, the ‘sienna 3’ module and the dark magenta module were highly related, and the yellow module and the purple module were highly related. However, their mutual correlations with each other were much stronger than their correlations with JA or SA (Figure 2a,c). Therefore, we chose the modules that had the strongest correlations with JA or SA as further research objects. The dark orange module included 162 genes and was referred to as the JA-related module; the ‘sienna 3’ module included 58 genes and was referred to as the bound SA-related module; and the yellow module included 1891 genes and was referred to as the free SA-related module. The eigengene dendrogram showed that these three modules belonged to three different branches (Figure 2b), suggesting that the expression of genes in the three modules might not be closely related.

### 2.5. Eigengene Expression of JA- and SA-Related Modules

**Figure 3:**
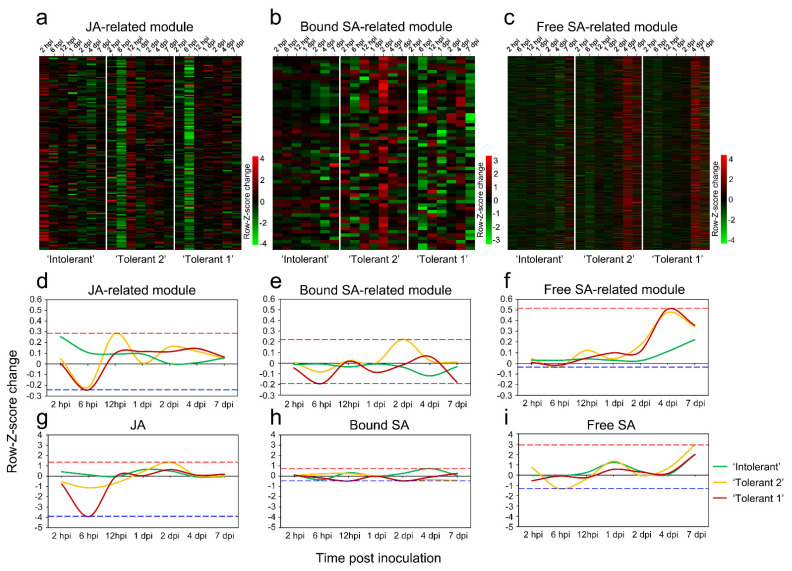
Gene expression patterns of JA- and SA-related modules. (**a**) Heatmap of JA-related module gene expression (rows, the Z-score was calculated for each gene, and the results are presented in rust-inoculated samples minus rust-free samples for each time point; the same for b and c) across the samples (columns; same for b and c). (**b**) Heatmap of bound SA-related module gene expression across the samples. (**c**) Heatmap of free SA-related module gene expression across the samples. (**d**) Comparison of eigengene expression (*y*-axis, the Z-score was calculated for the expression of each eigengene, and the results are presented in rust-inoculated samples minus rust-free samples for each time point; the same for e and f) in the JA-related module across the samples (*x*-axis, same for e and f). (**e**) Comparison of eigengene expression in the bound SA-related module across the samples. (**f**) Comparison of eigengene expression in the free SA-related module across the samples. (**g**) Comparison of the JA content (*y*-axis, the Z-score was calculated for level of each phytohormone, and the results are presented in rust-inoculated samples minus rust-free samples for each time point; the same for h and i) across the samples (*x*-axis; same for h and i). (**h**) Comparison of bound SA content across the samples. (**i**) Comparison of free SA content across the samples.

To further analyse the gene expression differences of JA- and SA-related genes in the three poplars at different time points, the expression patterns of all genes in the selected modules were compared. For each of the selected modules, an expanded view of the expression of all genes in the module was compared with the ME expression of the module across all samples. Z-scores were calculated for each row (each gene) and these were plotted instead of the normalized expression values (Figure 3a–c). The ME took on low values in arrays where many module genes were underexpressed (green colour in the heatmap). The ME took on high values in arrays where many module genes were overexpressed (red in the heatmap).

The ME values of each selected module were compared across the samples. The results showed that after E4 infection, the genes in the JA-related module and free SA-related module changed more than those in the bound SA-related module (Figure 3d,e). Correspondingly, the variation ranges of JA and free SA contents were larger than that of bound SA (Figure 3g–i). The most obvious difference among the three poplars was that in ‘Tolerant 1’ and ‘Tolerant 2’, the expression of most genes was unchanged or downregulated, while in ‘Intolerant’, the expression of most genes was upregulated in the early stage of infection (Figure 3d). Correspondingly, compared with ‘Intolerant’, the JA content in ‘Tolerant 1’ and ‘Tolerant 2’ in the early stage of infection showed a significant decrease (Figure 3g). These results suggested that JA and free SA-related genes should play a more important role in the response of poplar to E4 infection, and the increase in the expression of JA-related genes in the early stage of infection might affect the occurrence of defence responses by increasing the JA content, resulting in intolerance.

### 2.6. Expression Analysis of Plant-Pathogen Interaction-Related and Signalling-Related Genes in JA- and SA-Related Modules

Gene Ontology (GO) enrichment and Kyoto Encyclopedia of Genes and Genomes (KEGG) enrichment analyses were performed on the genes in the JA- and SA-related modules to identify the functions of these genes and the pathways that might be involved. GO enrichment analysis revealed that genes in these three selected modules were mainly enriched in catalytic activity (GO:0003824) and binding (GO:0005488) for the molecular function and in cell (GO:0005623), cell part (GO:0044464), membrane (GO:0016020), and membrane part (GO:0044425) for the cellular component (Figures S5–S7). Based on the KEGG enrichment analysis, genes in these three selected modules were mainly enriched in metabolic pathways (ko01100) and biosynthesis of secondary metabolites (ko01110, Tables S3–S5 and Figures S8–S10). Many genes in the JA- or free SA-related module were enriched in plant hormone signal transduction (ko04075, Tables S3 and S5). In addition, there were many genes enriched in plant–pathogen interaction (ko04626) and MAPK signalling pathway–plant (ko04016) in the free SA-related module (Table S5). Pathways associated with plant–pathogen interactions and signalling are detailed in Table S6. In general, compared with JA and bound SA, free SA was associated with more plant–pathogen interaction-related and signalling-related genes, indicating that in the response of poplar to E4 infection, free SA might participate in more regulatory pathways related to plant–pathogen interactions and signalling.

**Figure 4:**
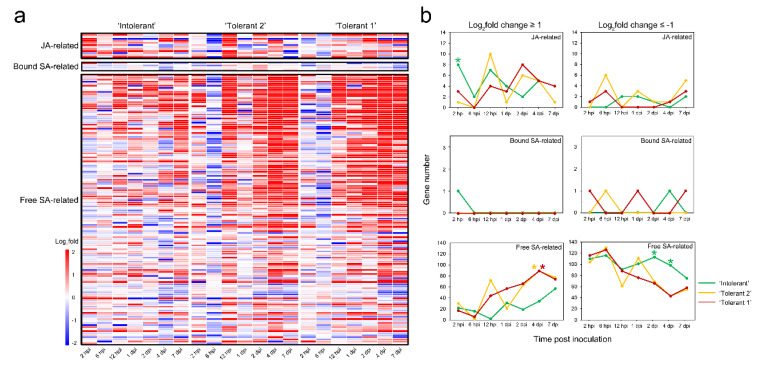
Expression analysis of plant–pathogen interaction-related and signalling-related genes in JA- and SA-related modules. (**a**) Heat map of plant–pathogen interaction-related and signalling-related genes in JA- and SA-related modules. (**b**) Count of differentially expressed plant–pathogen interaction-related and signalling-related genes in JA- and SA-related modules. The * indicates that the gene number is significantly higher.

The expression of these plant–pathogen interaction-related and signalling pathway-related genes at different time points after E4 infection was analysed, and it was found that in the tolerant poplars, the most significant common feature was that most of the genes related to JA were downregulated at 6 hpi and most of the genes related to free SA were upregulated at 12 hpi or 2 to 4 dpi (Figure 4). On the other hand, in ‘Intolerant’, most of the genes related to JA were upregulated at 2 hpi, while most of the genes related to free SA were downregulated at 2 dpi or 4 dpi (Figure 4). These results indicated that the expression of SA-related genes in the later stages of E4 infection might be a kind of control switch to start disease tolerance. However, the high expression of JA-related genes in the early stage of E4 infection might play an important role in distinguishing intolerance.

In addition, ‘Tolerant 1’ and ‘Tolerant 2’ had different gene expression patterns at 12 hpi or 1 dpi (Figure 4). In ‘Tolerant 1’, significantly more genes related to free SA were upregulated at 1 dpi, while in ‘Tolerant 2’, more genes related to JA were upregulated at 12 hpi. These gene expression changes should lead to subsequent changes in JA and free SA levels at the next time point (Figure 1b). This indicated not only that ‘Tolerant 1’ and ‘Tolerant 2’ should adopt different strategies in terms of dealing with E4 infection but also that the difference in the levels of JA and SA might be one of the reasons for the difference in tolerance to E4. More upregulated expression of free SA-related and less upregulated expression of JA-related plant–pathogen interaction and signalling-related pathway genes were associated with a more vigorous defence response in response to E4 infection.

### 2.7. Gene Network of JA- and SA-Related Modules

Although the expression changes of many plant–pathogen interaction-related and signalling-related genes were thought to be related to JA and SA, the contribution of each gene must be different and would play a different role in the response of poplar to E4 infection. Therefore, we further analysed the attributes of each gene in these JA- and SA-related modules. To quantify the similarity of genes in the selected modules, the associations of individual genes with different traits were quantified by defining gene significance (GS) as the correlation between the gene and the different traits. A quantitative measure of module membership (MM) was also defined as the correlation of the ME and the gene expression profile for the module. Scatterplots of GS vs. MM in the selected modules were plotted, and GS and MM were highly correlated, illustrating that genes highly significantly associated with a trait were often also the most important (central) elements of modules associated with the trait (Figure S11).

**Figure 5:**
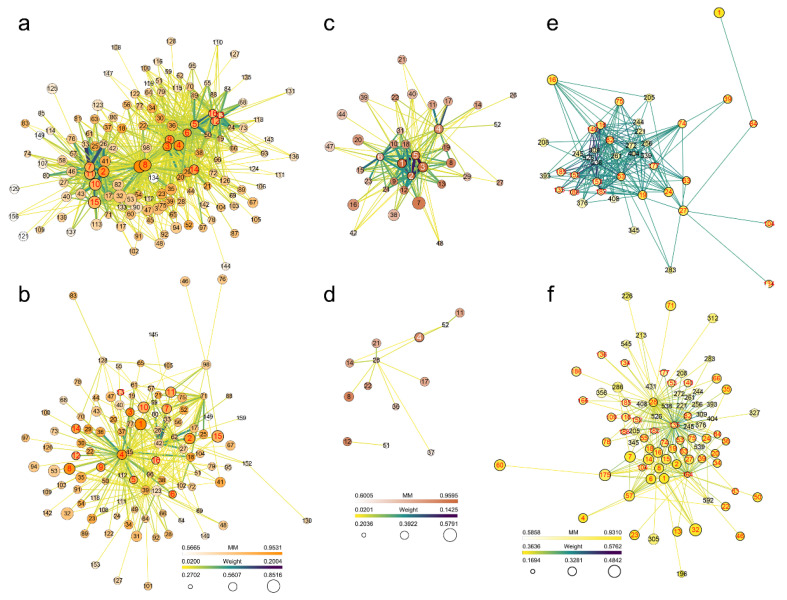
WGCNA revealed gene networks in the JA- and SA-related modules. (**a**) Gene network for hub genes in the JA-related module. (**b**) Gene network for plant–pathogen interaction-related and signalling-related genes in the JA-related module. (**c**) Gene network for hub genes in the bound SA-related module. (**d**) Gene network for plant–pathogen interaction-related and signalling-related genes in the bound SA-related module. (**e**) Gene network for hub genes in the free SA-related module (only genes with the top 200 highest weight values are shown). (**f**) Gene network for plant–pathogen interaction-related and signalling-related genes in the free SA-related module (only genes with the top 200 highest weight values are shown). The size of the dots represents GS. The colour of the dots represents MM. The colours and thicknesses of the lines represent the weight value between two genes. The labels of dots are listed based on IC, and the hub genes are highlighted with red or white colour labels. GS, gene significance; MM, module membership; IC, intramodular connectivity.

The intramodular connectivity (IC) for each gene in the selected modules was calculated. IC may be interpreted as a measure of MM. Genes with the top 10% highest IC values were considered intramodular hub genes in this study (Tables S7–S9), and they were thought to be the key controlling genes in the module. There were 16 hub genes in the JA-related module and 793 regulatory relationships between them and the other 123 genes. There were six hub genes in the bound SA-related module and 148 regulatory relationships between them and the other 30 genes. There were 189 hub genes in the free SA-related module and 180,664 regulatory relationships between them and the other 1368 genes. In the JA-related, bound SA-related and free SA-related modules, 12, four, and 132 genes were enriched in pathways associated with plant–pathogen interactions and signalling, respectively, as detailed in Tables S10–S12. These genes in these selected modules constitute 260, 12, and 60,203 regulatory relationships with the other 99, 10, and 1757 genes, respectively. The network data were exported to Cytoscape by Prefuse Force Directed Layout based on the weight value between two genes (Figure 5 and Figure S12). The results showed that some plant–pathogen interaction-related and signalling-related genes were also hub genes in the module, which should have regulatory relationships with more genes in the module. However, the GS values of some plant–pathogen interaction-related and signalling-related genes were not necessarily high, which should be because the content of JA or SA can be regulated by multiple genes.

Eight hub genes in the JA-related module were enriched in 13 pathways (Table S13). These genes were mainly enriched in pathways of metabolic pathways (ko01100) and biosynthesis of secondary metabolites (ko01110). The other pathways were all enriched by one gene, including plant–pathogen interaction (ko04626). One hub gene in the bound SA-related module was enriched in three pathways (Table S14), including ether lipid metabolism (ko00565), sphingolipid metabolism (ko00600), and metabolic pathways (ko01100). Sixty-one hub genes in the free SA-related module were enriched in 40 pathways (Table S15). These genes were mainly enriched in pathways of metabolic pathways (ko01100) and biosynthesis of secondary metabolites (ko01110). In addition, MAPK signalling pathway—plant (ko04016), starch and sucrose metabolism (ko00500), plant–pathogen interaction (ko04626), and plant hormone signal transduction (ko04075) were enriched by eight, seven, seven and five genes, respectively. Therefore, these plant–pathogen interaction-related and signalling-related genes likely play a dominant role in JA- or SA-related defence responses.

In the JA-related module, bound SA-related module, and free SA-related module, among the genes enriched in plant–pathogen interaction-related and signalling-related pathways and the genes they might relate to, a total of 49 genes were enriched in 45 pathways (Figure S13), a total of nine genes were enriched in 11 pathways (Figure S14), and a total of 634 genes were enriched in 120 pathways (Figure S15), respectively. The commonality of these genes from the three modules was that most of the enriched pathways belonged to global and overview maps. In addition, carbohydrate metabolism, signal transduction, environmental adaptation, lipid metabolism, and amino acid metabolism were all enriched by a large proportion of genes. These results suggested that plant–pathogen interaction-related and signalling-related genes in these hormone-related modules were involved in pathways with similar functions.

### 2.8. Gene Screening Based on Expression Differences

In this study, differentially expressed genes (DEGs) were defined by default as those with a false discovery rate (FDR) ≤ 0.05 and differences of more than twofold (log_2_fold change ≥1 or ≤−1). The hub genes, plant–pathogen interaction-related and signalling-related genes and genes that might be regulated by plant–pathogen interaction-related and signalling-related genes were screened for genes with significant differences in expression levels at different time points of E4 infection. We focused on genes with different expression trends in the three poplars.

In the JA-related module, 18 plant–pathogen interaction-related and signalling-related genes had obvious differences in expression changes among the three different poplars (Figure S16 and Table S16). Compared with the two tolerant breeds, especially ‘Tolerant 1’, 11 of these genes were upregulated at 2 hpi only in ‘Intolerant’ (Figure S16a–c and Table S16); nine of them were not upregulated or significantly downregulated at 2 dpi and/or 4 dpi only in ‘Intolerant’ (Figure S16d–f and Table S16), and another four genes were upregulated at 4 dpi only in ‘Intolerant’ (Figure S16g–i and Table S16). Therefore, JA-related genes were mostly upregulated in the early stage of infection and downregulated after the colonization of E4 in ‘Intolerant’.

**Figure 6:**
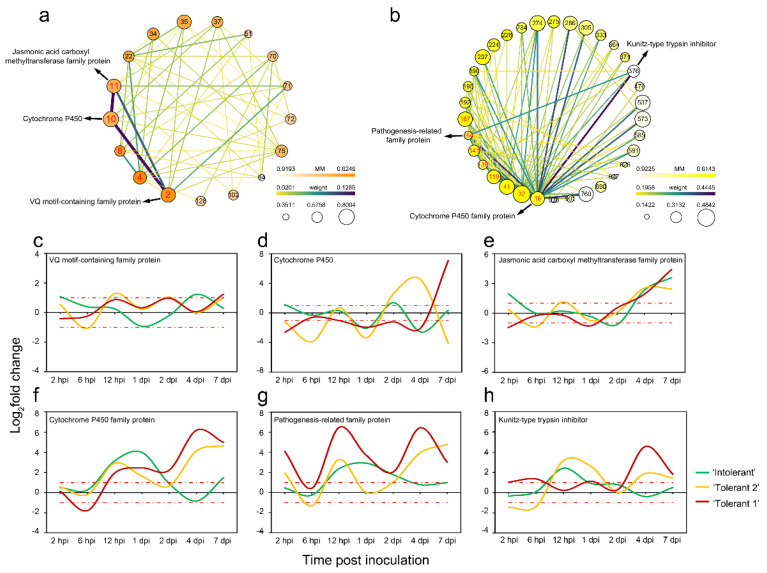
Gene networks for differentially expressed plant–pathogen interaction-related and signalling-related genes and highly related expression characteristics. (**a**) Gene network for differentially expressed plant–pathogen interaction-related and signalling-related genes in the JA-related module. (**b**) Gene network for differentially expressed plant–pathogen interaction-related and signalling-related genes in the free SA-related module (only genes with the top 100 highest weight values are shown). The size of the dots represents GS. The colour of the dots represents MM. The colours and thicknesses of the lines represent the weight value between two genes. The labels of dots are listed based on IC, and the hub genes are highlighted with red labels. (**c**–**h**) Comparison of the expression changes of highly related genes among the three poplars. GS, gene significance; MM, module membership; IC, intramodular connectivity.

In the JA-related module, the gene network of these plant–pathogen interaction-related and signalling-related genes suggested that they might regulate each other (Figure 6a). Based on the NR (nonredundant proteins) database, the functions of the proteins encoded by those genes were annotated with BLASTP. Among them, a hub gene homologous to LysM domain-containing receptor-like kinase 4 family protein (XP_002321141.2, No. 4) had the most regulatory relationships, and its most significant expression feature was that compared with ‘Tolerant 2’ and ‘Tolerant 1’, the gene was not upregulated at 2 dpi and 4 dpi in ‘Intolerant’. In addition, genes homologous to targets of Myb protein 1-like (XP_011006301.1, No. 22) or the S-adenosylmethionine synthase family protein (XP_002314993.1, No. 75) had the second most regulatory relationship, and their common significant expression feature was that compared with ‘Tolerant 2’ and ‘Tolerant 1’, these genes were upregulated at 2 hpi only in ‘Intolerant’.

In addition, strong correlations were found among three hub genes (Figure 6a) in the JA-related module, which were homologous to the VQ motif-containing family protein (XP_002307385.1, No. 2), cytochrome P450 (AHF20912.1, No. 10), and jasmonic acid carboxyl methyltransferase family protein (XP_002307671.1, No. 11). The validation of expression profiles of these genes was verified by quantitative real time polymerase chain reaction (RT-qPCR). These three hub genes were all upregulated at 2 hpi only in ‘Intolerant’ (Figure 6c–e and Figure S17a–c), and their GS values were relatively high, suggesting that they might play an important role in the increase in JA levels at 2 hpi by regulating each other.

In the bound SA-related module, three plant–pathogen interaction-related and signalling-related genes had obvious differences in expression changes among the three different poplars (Figure S18 and Table S17). Among these genes, two were homologous to the NBS-LRR resistance gene-like protein ARGH30 (XP_006388824.1, No. 1 and No. 5) and were significantly downregulated at 4 dpi in ‘Intolerant’ (Figure S18a–c and Table S17). They were both hub genes and might regulate each other. Another gene was homologous to the cytochrome P450 family protein (XP_002319566.2, No. 37) and was upregulated at 4 dpi only in ‘Intolerant’ (Figure S18d–f and Table S17). These results indicated that the expression changes of some *R* genes might be affected by changes in the bound SA content, and the downregulation of these *R* genes at 4 dpi might be related to the intolerance of poplar to E4.

In the free SA-related module, 85 plant–pathogen interaction-related and signalling-related genes had obvious differences in expression changes among the three different poplars (Figure S19 and Table S18). Among these genes, 58 were not upregulated or were significantly downregulated at 4 dpi only in ‘Intolerant’ (Figure S19a–c and Table S18), 13 were upregulated at 2 hpi only in ‘Intolerant’ (Figure S19d–f and Table S18), 11 were downregulated at 1 dpi and/or 2 dpi only in ‘Intolerant’ (Figure S19g–i and Table S18), and another seven genes were upregulated at multiple time points from 6 hpi to 4 dpi in ‘Intolerant’ (Figure S19j–l and Table S18). These results showed that free SA-related genes were mostly downregulated when E4 finished colonization in ‘Intolerant’.

In the free SA-related module, the gene network of these plant–pathogen interaction-related and signalling-related genes suggested that they might regulate each other (Figure 6b). Among these genes, a hub gene homologous to PREDICTED: cytochrome P450 87A3-like (XP_011007351.1, No. 41) had the most regulatory relationships, and its most significant expression feature was that the gene was not upregulated at 4 dpi only in ‘Intolerant’. Other hub genes homologous to the cytochrome P450 family protein (XP_002306491.1, No. 16), K^+^ rectifying channel family protein (XP_002324257.1, No. 32), Kunitz-type trypsin inhibitor (ADW95385.1 and ADW95376.1, No. 119 and 139), pathogenesis-related family protein (XP_006379156.1 and XP_002300292.2, No. 141 and 164), or allene oxide synthase family protein (XP_002305405.1, No. 187) also had more regulatory relationships. In addition, genes homologous to the calcium-binding family protein (XP_002306659.1, No. 196), putative cytochrome P450 family protein (XP_006375029.1, No. 192), or cytochrome P450 family protein (XP_002309865.2, No. 234) had more regulatory relationships. Among these genes, the gene homologous to the calcium-binding family protein was upregulated at 2 hpi only in ‘Intolerant’, and the other genes were not upregulated or had much lower expression levels at 4 dpi only in ‘Intolerant’. These results indicated that the plant–pathogen interaction-related and signalling-related genes associated with free SA were significantly inhibited in ‘Intolerant’, including some *PR* genes. This inhibition occurred mainly when E4 completed colonization.

Strong correlations were found among the three hub genes (Figure 6b) in the free SA-related module, which were homologous to the cytochrome P450 family protein (XP_002306491.1, No. 16), pathogenesis-related family protein (XP_002300292.2, No. 164) and Kunitz-type trypsin inhibitor (ADW95389.1, No. 376). The validation of expression profiles of these genes was verified by RT-qPCR. These three hub genes were also not upregulated at 4 dpi only in ‘Intolerant’. However, in ‘Tolerant 1’ and ‘Tolerant 2’, these genes were significantly upregulated at 4 dpi, when multiple chlorotic spots appeared on the leaves (Figure 6f–h and Figure S17d–f). These results suggested that these genes were severely suppressed during E4 colonization in ‘Intolerant’ and might be related to defence responses in ‘Tolerant 1’ and ‘Tolerant 2’.

In addition, some of the genes homologous to the cytochrome P450 family protein were related to JA, some were related to SA, and they were all hub genes of the modules, suggesting that cytochrome P450 family genes likely play a dominant role in JA- and/or SA-related pathways during the process of poplar defence against E4.

### 2.9. Identification of Genes Related to JA and SA Biosynthesis and Defence-Related Genes Downstream of JA and SA

Based on the results of a comparison with the NR database and the results of DEG analysis, we found that two genes homologous to NBS-LRR (XP_006388824.1) were correlated with bound SA and downregulated at 4 dpi only in ‘Intolerant’ (Figure S20a,b). Another gene homologous to NBS-LRR (ABF81442.1) was correlated with free SA and was not upregulated until 4 dpi in ‘Intolerant’. However, in ‘Tolerant 2’, the gene was upregulated at 12 hpi and continued to be upregulated until 4 dpi. In ‘Tolerant 1’, the gene began to be upregulated immediately at 2 hpi, and except for a downregulation at 6 hpi, the expression levels remained high at other time points (Figure S20c). The above results indicated that the expression of some *R* genes was related to SA, and the expression of these *R* genes was delayed or inhibited in ‘Intolerant’ after E4 infection.

A gene homologous to enhanced disease susceptibility 1 (EDS1, XP_002322142.1) was correlated with free SA. The gene continued to be upregulated from 12 hpi to 7 dpi in ‘Tolerant 1’. In ‘Tolerant 2’, the gene was also upregulated at 12 hpi, but it showed no obvious change at 1 dpi and then its expression was upregulated again from 2 dpi to 7 dpi. However, in ‘Intolerant’, even though the gene was upregulated at 12 hpi, 4 dpi, and 7 dpi, it showed no obvious change at either 1 dpi or 2 dpi (Figure S20d). Therefore, EDS1 might need to be expressed continuously to ensure the transmission of SA-related defence signals. We also found that a gene homologous to non-race-specific disease resistance 1 (NDR1, XP_002317572.1) was correlated with free SA. The expression levels of the gene did not change significantly at multiple time points in all three poplars, except at 4 dpi, when it had the only obvious upregulation in ‘Tolerant 2’, and it was also upregulated slightly in ‘Tolerant 1’, but it was slightly downregulated in ‘Intolerant’ (Figure S20e). These results indicated that the NDR1-related SA-related defence signalling pathway was also inhibited in ‘Intolerant’ at 4 dpi. At this time point, the tolerant poplars had already begun to show obvious defence responses, which suggested that *NDR1* might be associated with the signalling of defence responses in poplars against E4.

Three genes homologous to WRKY 72 (XP_011047157.1), WRKY47 (XP_002302808.1), and WRKY 65 (XP_002317397.1) were found to be correlated with free SA. The distinguishing feature of these genes was that their expression levels in ‘Intolerant’ were significantly lower than those in ‘Tolerant 2’ and ‘Tolerant 1’ at 4 dpi. In addition, compared with ‘Tolerant 2’ and ‘Tolerant 1’, the expression level of *WRKY47* did not change significantly at all time points in ‘Intolerant’ (Figure S20f–h). It is possible that SA-related defence signalling pathways were not activated upstream of WRKY at 4 dpi in ‘Intolerant’, and therefore, the expression of *WRKY*s was fully suppressed at this time point.

Four genes with high homology to four PRs (XP_006379156.1, XP_002300292.2, XP_002306682.1, and XP_002313936.2) were also found to be correlated with free SA. Compared with their expression in ‘Tolerant 2’ and ‘Tolerant 1’, these four homologous *PR*s showed downregulation or no obvious change in ‘Intolerant’, which started at 2 hpi at the earliest and could last up to 7 dpi, indicating that the expression of *PR*s in ‘Intolerant’ was not regulated or suppressed (Figures S17e and S20i–k). Therefore, the expression of *PR*s, which are downstream of the SA-related defence signalling pathway, was inhibited in ‘Intolerant’. This was probably also due to suppression of the expression of their upstream genes.

Most phytohormone-related genes were only upregulated in ‘Intolerant’ at 2 hpi. Among them, a gene homologous to jasmonic acid carboxyl methyltransferase family protein (JACM, XP_002307671.1), which catalyses the methylation of jasmonate into methyl jasmonate, was highly correlated with JA and upregulated at 2 hpi only in ‘Intolerant’ (Figure S20l). Another gene homologous to the lipoxygenase family protein (LOX, XP_006369133.1), which catalyses the initial step of JA synthesis, was also highly correlated with JA and upregulated at 2 hpi only in ‘Intolerant’ (Figure S20m). These two genes might account for the higher JA content of ‘Intolerant’ at 2 hpi (Figure 1b).

In addition, we found that some JA biosynthesis-related genes were correlated with free SA. For example, a gene homologous to the allene oxide synthase family protein (AOS, XP_002305405.1), which catalyses the first committed step of JA biosynthesis, was correlated with free SA, and its expression level in ‘Intolerant’ was much lower than that in ‘Tolerant 2’ and ‘Tolerant 1’ at 4 dpi (Figure S20n), which might account for the lower JA content of ‘Intolerant’ at 4 dpi (Figure 1b). This suggested that JA levels were also affected after E4 colonization and that this change might be regulated by SA. In addition, a gene homologous to allene oxide cyclase 3, chloroplastic-like (AOC, XP_011021732.1), which is involved in the production of 12-oxo-phytodienoic acid, a precursor of JA, was correlated with the free SA content and was upregulated at 2 hpi only in ‘Intolerant’ (Figure S20o), which might also account for the higher JA content of ‘Intolerant’ at 2 hpi (Figure 1b). These results suggested that JA might play an important role in the early period of E4 infection, but the content of JA decreased in the later period of E4 infection, which might be beneficial to the propagation and colonization of E4.

**Figure 7:**
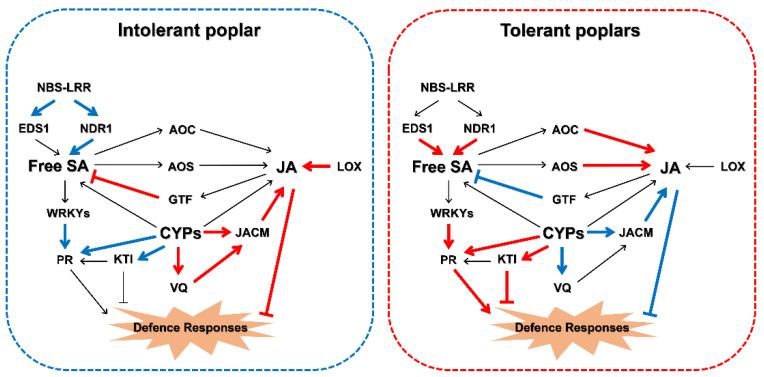
Model of how plant–pathogen interaction-related and signalling-related genes may interfere with E4 infection in tolerant and intolerant poplars. The bold red indicates that gene expression is specifically upregulated at a specific time point, and the bold blue indicates that gene expression is specifically downregulated at a specific time point. This specific time point is usually 2 hpi or 6 hpi for JA-related genes and 2 dpi or 4 dpi for SA-related genes. Normal black indicates that there is no significant difference in gene expression between the three poplars. NBS-LRR, NBS-LRR resistance gene-like protein; EDS1, enhanced disease susceptibility 1 family protein; NDR1, non-race-specific disease resistance 1 family protein; WRKY, WRKY transcription factor; PR, pathogenesis-related family protein; AOC, allene oxide cyclase 3, chloroplastic-like; AOS, allene oxide synthase family protein; LOX, lipoxygenase family protein; GTF, putative glucosyltransferase family protein; CYP, cytochrome P450 family protein; JACM, jasmonic acid carboxyl methyltransferase family protein; KTI, Kunitz-type trypsin inhibitor; VQ, VQ motif-containing family protein.

We also found that a gene homologous to the putative glucosyltransferase family protein (GTF, XP_002306205.2), which might be involved in the accumulation of glucosyl SA during pathogenesis, was highly correlated with JA. The gene continued to be highly expressed from 12 hpi to 7 dpi in ‘Tolerant 1’ and was downregulated at 2 hpi to 6 hpi but upregulated at 12 hpi and 7 dpi in ‘Tolerant 2’. However, it was only upregulated at 6 hpi and 12 hpi in ‘Intolerant’, and there was no significant change in the amount of expression thereafter, which might account for the sudden increase in bound SA in ‘Intolerant’ from 6 hpi to 12 hpi (Figure S20p). The validation of expression profiles of these selected genes was verified by RT-qPCR (Figure S20). This suggested that JA might be associated with the conversion of SA. Therefore, in the process of the poplar response to E4 infection, SA and JA should be able to affect changes in the content of each other and ultimately affect the expression of plant–pathogen interaction-related and signalling-related genes (Figure 7). This effect might be manipulated by E4, which determined the susceptibility of different poplars.

## 3. Discussion

In this study, we correlated the gene expression of two tolerant poplars and an intolerant poplar with changes in two important plant–pathogen interaction-related and signalling-related phytohormones, JA and SA, after E4 inoculation. In our results, we found a significantly higher number of plant–pathogen interaction-related and signalling-related genes associated with free SA. This suggested that in response to E4 infection, most of the plant–pathogen interaction-related and signalling-related genes might be influenced by SA signalling and ultimately determine whether to develop a defence response. We also found that in response to E4 infection, both tolerant poplars and intolerant poplar needed to go through two stages. The first was the synthesis and accumulation of JA and SA, which then activated the downstream signalling pathway and induced the expression of various defence-related genes. During the infection process, however, the expression of genes in each pathway in these poplars was affected by E4 to varying degrees. First, the decrease in JA production gene expression and JA content in the early stage of infection should be beneficial to the occurrence of tolerance reactions. Second, the SA pathway is a kind of control switch to start disease tolerance. The key divergence between tolerant and intolerant poplar was the blocking of signal conduction in the first stage (EDS1 and NDR1). The blockage led to the difference in SA levels, caused the differences in gene expression in the next stage and led to the final phenotypic differences, such as delayed growth and reproduction of E4. This series of differences was one of the primary reasons for tolerance to maintain maximum viability of the leaves. Third, ‘Tolerant 2’ and ‘Tolerant 1’ adopted different strategies in terms of free SA-related tolerance, although both poplars could inhibit and delay the growth and reproduction of E4 to a certain extent. Finally, increasing JA levels in the early stage could lead to the inhibition of SA-related defence signalling.

The major stages of *M. larici-populina* infection include germination and penetration, early colonization of plant tissue, colonization of the plant mesophyll, and uredinia formation [52,53]. We found that the expression of some *R* genes was related to SA and was delayed or inhibited when dense networks of infection hyphae and haustoria formed in ‘Intolerant’ [54,55]. Therefore, the timely and sustained high expression of the *R* gene should play an important role in the defence of poplar against E4 infection. R proteins can be further divided into two groups according to the N-terminal sequence: coiled-coil (CC)-NBS-LRR and Toll-interleukin-1 receptor (TIR)-NBS-LRR [56]. In *Arabidopsis*, the *EDS1* and *NDR1* genes have been shown to encode essential components of race-specific disease resistance [57]. Some CC-NBS-LRR-type proteins are found to signal through NDR1 [57]. On the other hand, some TIR-NBS-LRR-type proteins functionally require EDS1 [57,58]. Both NDR1 and EDS1 are known to act upstream of SA to regulate SA accumulation [22,28,58,59,60]. SA first binds to an SA-binding protein (SABP), and then the “SA-SABP” complex transmits information to a secondary intracellular messenger, which is amplified by a self-feedback mechanism and then transduced inside the cell, triggering the HR. At the same time, SA will cause changes in the intracellular redox state, activate the activation and interaction of transcription factors such as nonexpressor of pathogenesis-related genes 1 (NPR1), TGA and WRKY, and ultimately induce the expression of PRs and the emergence of systemic acquired resistance (SAR) [61,62]. Overexpression of NDR1, EDS1, NPR1, or several other SA regulators confers enhanced disease resistance to a range of pathogens in *Arabidopsis* and/or in other plants [59,60,63,64,65,66,67,68,69].

Our results indicated that EDS1 plays an important role in signal transmission and may need to be expressed continuously to ensure the transmission of SA-related defence signals. The NDR1-related SA-related defence signalling pathway was inhibited in ‘Intolerant’ at 4 dpi, when the tolerant poplars had already begun to show obvious defence responses. Therefore, compared with that in the tolerant breeds ‘Tolerant 1’ and ‘Tolerant 2’, the expression of *R* genes and their downstream genes correlated with SA were not upregulated in time or were downregulated at critical time points in ‘Intolerant’. These results suggested that even though ‘Intolerant’ responded to the invasion of E4, E4 was able to overcome its plant surveillance systems, probably by secreting effectors to manipulate the plant defence system during its growth and colonization [54,55]. Our results also showed that although the expression of several genes in the SA-related defence signalling pathway was suppressed mainly at 4 dpi in ‘Intolerant’, suppression of the expression of *PR* genes appeared as early as 2 hpi, which suggested that there might be other genes that interfered with them [70,71].

In addition, we also found that the expression of some genes in the SA-related defence signalling pathway was inhibited in the early infection period of ‘Tolerant 1’ and ‘Tolerant 2’, and this inhibition mostly occurred at 6 hpi and 2 hpi, suggesting that E4 could also interfere with the defence responses of poplars with tolerance to E4, which was reflected in the decrease in the expression of multiple *PR* genes at 2 hpi or 6 hpi. It cannot be ignored that the JA content in ‘Intolerant’ was significantly increased at 2 hpi (Figure 1b), which might play an important role in manipulating the expression of *PR*s in ‘Intolerant’. Some JA synthesis-related genes were found to be correlated with free SA, suggesting that the change in JA levels after E4 colonization might be regulated through SA. However, JA was also found to be associated with the conversion of SA. Therefore, SA and JA should be able to affect content changes of each other and ultimately affect the expression of plant–pathogen interaction-related and signalling-related genes in the process of the poplar response to E4 infection.

In ‘Intolerant’, the content of free SA increased gradually from 12 hpi to 2 dpi and then decreased at 4 dpi when uredinia started to form. The content of the bound SA increased at 12 dpi and 4 dpi, which were determined to be representative time points of the infection phase and propagation phase of E4, respectively [54], indicating that SA did not completely fail to play a role in the process of defence against E4 infection in ‘Intolerant’, but the changes in its content and form might be inhibited after E4 colonization, and its defence signalling was suppressed at those crucial time points [54,55]. On the other hand, in ‘Tolerant 1’, the content of free SA did not increase until visible scattered lesions started to appear at 2 dpi, and it decreased when some necrosis gradually appeared but increased again when confluent necrosis appeared, suggesting that the content of free SA might be related to programmed cell death (PCD).

Although the SA and JA defence pathways occasionally cooperate, mostly they are mutually antagonistic, and this regulatory cross talk may have evolved to allow plants to fine-tune the induction of their defences in response to different plant pathogens [70,72,73], but pathogens may have also evolved to exploit this fact to overcome SA-mediated defence responses [50]. Therefore, we hypothesized that the success of E4 in infecting ‘Intolerant’ may be due to its ability to manipulate the content of plant hormones, such as increasing the JA content in the early infection stage and then interfering with the accumulation of SA and the expression of plant–pathogen interaction-related and signalling-related genes.

In addition, we found that *cytochrome P450 family* (*CYP*) genes play an important role in both the JA and SA pathways (Figure 6a,b). The CYP superfamily is the largest enzymatic protein family in plants, and its members are involved in multiple metabolic pathways with distinct and complex functions, playing important roles in plant defence through their involvement in phytoalexin biosynthesis, hormone metabolism, and the biosynthesis of some other secondary metabolites [74]. One of the CYP (AHF20912.1) homologous genes might interact with a JACM homologous gene and a VQ motif-containing family protein (hereafter referred to as VQ, XP_002307385.1) homologous gene, and these genes were both related to JA. Increasing evidence demonstrates the involvement of VQ proteins in SA- and/or JA-mediated defence responses [75]. The expression of the *VQ* gene was upregulated at 2 hpi and 4 dpi only in ‘Intolerant’, suggesting that the upregulation of this *VQ* gene might be beneficial to E4 infection. JACM is a key enzyme for jasmonate-regulated plant responses, which can enhance the resistance level of *Arabidopsis* against the virulent fungus *Botrytis cinerea* [76], which is a necrotrophic pathogen. The expression of the *JACM* gene was found to be upregulated at 2 hpi only in ‘Intolerant’; therefore, we hypothesized that the upregulation of this *JACM* gene may help the biotrophic pathogen E4 successfully infect ‘Intolerant’.

Another CYP family (XP_002306491.1) homologous gene might be involved in the regulation of a Kunitz-type trypsin inhibitor (KTI, ADW95389.1) homologous gene and a PR (XP_002300292.2) homologous gene, and these genes were all related to free SA. Individual PRs are induced to various extents by different signals, including SA and JA [77]. The mixture of signals released or produced upon microbial stimulation appears to determine the magnitude of the response of the plant and its effectiveness in inhibiting further infection. In *Arabidopsis,* SA-dependent expression of PR-1, PR-2 and PR-5 is required for increased protection against the biotrophic fungus *Peronospora parasitica*, whereas SA-independent but JA-dependent expression of PDF1.2, PR-3, and PR-4 is associated with induced resistance against necrotrophic fungi [77,78,79]. We hypothesized that E4 might manipulate the expression of SA-related *PR* gene expression in ‘Intolerant’ to promote its infection, as the expression of this *PR* gene was not upregulated at 2 hpi or 4 dpi only in ‘Intolerant’. The other gene, *KTI,* was reported to be involved in modulating PCD in plant–pathogen interactions and induced by pathogens and by SA [80]. KTI is a protease inhibitor (PI) that is specific for serine proteases. Plants possess a large arsenal of PIs that have been proposed to function as storage proteins and regulators of endogenous proteinases. It has been reported that *AtKTI1* triggered by pathogen-derived elicitors encodes a functional serine protease inhibitor and antagonizes pathogen-associated cell death in *Arabidopsis* [80]. We found that the expression of *KTI* was upregulated in ‘Intolerant’ at 12 hpi when E4 was in its early colonization period [52,53] and did not change obviously at other time points. In ‘Tolerant 2’, the expression level of *KTI* decreased at 2 hpi and 6 hpi but increased at 12 hpi, 1 dpi, 4 dpi, and 7 dpi. In ‘Tolerant 1’, the gene was upregulated at most time points, especially at 4 dpi and 7 dpi, when the observation of PCD became apparent. Therefore, KTI in poplar might act as a regulator in cellular defence responses, and its expression might be regulated by SA. The expression of the *KTI* gene was regulated to activate or inhibit the formation of PCD to help keep the plant in the best balance to protect itself to the greatest extent.

To sum up, in response to major stages of E4 infection, both tolerant poplars and intolerant poplar synthesised and accumulated JA and SA, then activated the downstream signalling pathway and induced the expression of various defence-related genes. The key divergence between tolerant and intolerant poplars was the blocking of signal conduction in the first stage. E4 should be able to successfully interfere with genes related to JA and SA biosynthesis and manipulate their levels in poplar during infection, thus affecting the expression of defence-related genes downstream of JA and SA and the eventual occurrence of defence responses (Figure 7). Finally, the increase in JA levels in the early stage could lead to the inhibition of SA-related defence signalling. These stages can exactly correspond to the major stages of *M. larici-populina* infection, including germination and penetration, early colonization of plant tissue, colonization of the plant mesophyll, and uredinia formation.

Tolerant poplars could promptly regulate the occurrence of defence responses by activating or inhibiting SA or JA pathways in a timely manner, including regulating the expression of genes related to PCD to limit the growth of E4 and protect themselves. In this process, *KTI* may play an important role and it may be regulated by a *CYP*. This suggests that these two genes may be used as research objects to study the mechanism of PCD during poplar-rust interaction. In contrast, in intolerant poplar, the inhibition of SA-related defence signalling through increasing JA levels in the early stage led to continued inhibition of a large number of plant–pathogen interaction-related and signalling-related genes, including *NBS-LRR*s, *EDS1*, *NDR1*, *WRKY*s, and *PR*s. These genes may be the key factors to determine whether poplar can produce defence responses in time, and the specific functions of these genes in the process of rust infection need to be verified in the future. In addition, because the changes in the expression of these genes were related to the changes in the content of JA or SA, it is also of great significance to study the effect of the changes in the expression of genes related to the synthesis of JA and SA on the improvement of poplar resistance against rust, which includes *AOC*, *AOS*, *GTF*, *LOX,* and *JACM*.

Therefore, timely activation or inhibition of the SA or JA pathways is the key difference between tolerant and intolerant poplars. In addition, this interference effect was not immutable. E4 adjusts the interference strategy according to the need for infection and maintains maximum viability of the leaves to successfully colonize and produce new urediniospores. However, in ‘Tolerant 1’ and ‘Tolerant 2’, although the genes in the SA-related defence signalling pathway might also be partly influenced by E4, the regulatory mechanism of poplars themselves played a leading role in the process of dealing with the infection. These mechanisms would be able to constantly adjust the expression patterns of the involved genes to facilitate the defence response in time to help the plant protect itself.

## 4. Materials and Methods

### 4.1. E4 Isolates and Plant Materials

E4-infected poplar leaves were collected from *P. trichocarpa* cv. Trichobel *at* Markington (northern England) [1], and rust isolates were derived from single uredinial pustules as previously reported [1,54,55]. The spores were stored at −20 °C. One-year-old hybrid poplars, an intolerant poplar *P. nigra* × *P. deltoides* (‘Intolerant’) [54,55,81], and two tolerant poplars, *P. deltoides* × *P. trichocarpa* (‘Tolerant 2’) and *P. trichocarpa* × *P. deltoides* (‘Tolerant 1’), were used as the sources of plant tissue. These hybrid poplars were grown as described previously [54,55].

### 4.2. Inoculation Procedure and Total RNA Preparation

Leaves of hybrid poplars were inoculated with E4 as described by Pei et al. [1] and Chen et al. [54,55] and incubated in a phytotron at 16 °C with 16 h day^−^^1^ illumination (80 μE m^−2^ s^−1^) for different periods as described before (2 h, 6 h, 12 h, 1 d, 2 d, 4 d, and 7 d). Control groups contained E4-free leaves (leaves were treated with deionized water only) incubated under the same conditions. Disease was scored based on pustule area and inoculum density data according to the method described by Pei et al. [1,82,83] and Chen et al. [55]. Each leaf sample was then frozen and ground using liquid nitrogen and divided into two parts, one for RNA extraction and one for SA and JA determination.

### 4.3. Total RNA Preparation and DGE Library Preparation and Sequencing

The cetyltrimethylammonium bromide (CTAB) method was used to extract total RNA from frozen leaves [54,55]. RNA was extracted by using organic solvents after CTAB combined with proteins and polysaccharides. After removing impurities such as protein, polysaccharides, and phenols, ethanol was added and RNA was precipitated and separated. The RNA Nano 6000 Assay Kit (Agilent Technologies, Santa Clara, CA, USA) of the Agilent Bioanalyzer 2100 system (Agilent Technologies, Santa Clara, CA, USA) was used to assess RNA quality. The purity of RNA was determined by a NanoDrop^TM^ spectrophotometer (Thermo Scientific, Wilmington, DE, USA). Genomic DNA was removed using DNase I, Amplification Grade (Invitrogen, Foster, CA, USA, cat. no. 18068-015). Each RNA sample was sheared and reverse transcribed using random primers to obtain cDNA for library construction. The construction of the libraries and sequencing were all performed on a BGISEQ-500 RNA-seq platform (BGI, Shenzhen, China), and 50-bp single-end (SE) reads were generated. SOAPnuke (v1.5.6, https://github.com/BGI-flexlab/SOAPnuke, accessed on 24 November 2016) was used to filter the generated raw sequencing reads. Clean reads were mapped to the reference genome *P. trichocarpa* (version 3.0, http://www.phytozome.net/poplar.php, accessed on 26 November 2018) using HISAT2 (v2.0.4, http://daehwankimlab.github.io/hisat2, accessed on 18 May 2016) [84,85]. Clean reads were then mapped to reference sequences using Bowtie2 (v2.3.4.2, http://bowtie-bio.sourceforge.net/Bowtie/index.shtml, accessed on 7 August 2018) [86]. The matched reads were calculated and normalized to fragments per kilobase per million mapped fragments (FPKM) using RSEM (v1.2.12, http://deweylab.biostat.wisc.edu/RSEM, accessed on 27 May 2014) [87].

### 4.4. Quantification of SA and JA Levels

SA and JA were quantified by high performance liquid chromatography (HPLC) mass spectrometry from crude plant extracts according to the method of Pan et al. [88]. SA or JA was extracted and quantified using an ultra-HPLC-Q-Exactive^TM^ system (Thermo Scientific, San José, CA, USA) using an ODS column (μ-Bondasphere C18, 5 μm, 3.9 mm × 150 mm, 100A; Waters, Milford, MA, USA) or an ODS column (AQUITY UPLC BEH C18, 1.7 μm, 2.1 mm × 100 mm; Waters, Milford, MA, USA) as described [88]. Authentic SA and JA (Sigma Aldrich, Burlington, MA, USA and Olchemim Ltd., Olomouc, Czech Republic) were used as external standards. Three separate biological replicates of each treatment were performed, and each replicate was assessed three times.

### 4.5. WGCNA

WGCNA was used to explore the complex relationships between genes and phenotypes (https://horvath.genetics.ucla.edu/html/CoexpressionNetwork, accessed on 7 August 2018) [51]. The appropriate power value was determined when the degree of independence exceeded 0.8. The minimum number of genes was set as 30 for the high reliability of the results. The module eigengene (ME) is defined as the first principal component of a given module, which can be considered representative of the gene expression profiles in a module. Module-trait associations were estimated using the correlation between the ME and the trait. The intramodular connectivity (IC) was calculated for each gene by summing the connection strengths with other module genes and dividing this number by the maximum intramodular connectivity. The IC value was defined only for the genes inside a given module. IC measures how connected or coexpressed a given gene is with respect to the genes of a particular module. For each expression profile, gene significance (GS) was calculated as the absolute value of the Pearson correlation between the expression profile and each trait. Module membership (MM) was defined as the Pearson correlation of the expression profile and each ME. Network depictions were constructed with Cytoscape software [89]. Gene Ontology (GO) enrichment [90] and Kyoto Encyclopedia of Genes and Genomes (KEGG) enrichment analysis were performed on selected genes [91]. The results of the analyses were extracted, and a *p*-value ≤ 0.05 after the correction was used as the threshold. The top 10 records were extracted if there were more than 10 records. The depictions were constructed using the OmicShare tools, a free online platform for data analysis (http://www.omicshare.com/tools, accessed on 7 August 2018).

### 4.6. RT-qPCR

RT-qPCR was performed using the GoTaq 2-Step RT-qPCR System Kit (Promega, Madison, WI, USA, catalogue number: A6010) and a Stratagene Mx3000P qPCR system (Agilent Technologies, Santa Clara, CA, USA) according to the instructions of the manufacturer. Primers used in this study are listed in Table S19. The RT-qPCR results were normalized as log_2_fold changes with a constitutively expressed gene, *18S ribosomal RNA*, as an internal control. The $2^{-\Delta \Delta C_{T}}$ method was used to analyse the data [54,55]. The experiments were repeated three times. Significance was determined by Student’s *t*-test.

## 5. Conclusions

Tolerant poplars could promptly regulate the occurrence of defence responses by activating or inhibiting JA and SA pathways in a timely manner, including the expression of downstream genes, to protect themselves. In contrast, in intolerant poplar, the inhibition of SA-related defence signalling through increasing JA levels in the early stage led to continued inhibition of a large number of plant–pathogen interaction-related and signalling-related genes. Therefore, timely activation or inhibition of the JA and SA pathways is the key difference between tolerant and intolerant poplars.

## Data Availability

The data presented in this study are available in Supplementary Material.

## Supplementary Materials

The following are available online at https://www.mdpi.com/article/10.3390/ijms22095001/s1.
