# Supplementary material for: Integrating Transcriptome and Coexpression Network Analyses to Characterize Salicylic Acid- and Jasmonic Acid-Related Genes in Tolerant Poplars Infected with Rust
Source: Int J Mol Sci. 2021 May 8;22(9):5001. doi: 10.3390/ijms22095001 (PMC8125932; doi:10.3390/ijms22095001)
Supplement: Supplementary file 1 [file ijms-22-05001-s001.zip › ijms-1189629-supplementary.pdf]

Integrating Transcriptome and Coexpression Network Analyses to Characterize Salicylic Acid- and Jasmonic Acid-Related Genes in Tolerant Poplars Infected with Rust

Qiaoli Chen [1,2,†], Ruizhi Zhang [1,†], Danlei Li [1,2], Feng Wang [1,2,*]

1 Key Laboratory of Alien Forest Pests Detection and Control-Heilongjiang Province, School of Forestry, North-east Forestry University, Harbin 150040, China

2 Key Laboratory of Sustainable Forest Ecosystem Management-Ministry of Education, Northeast Forestry Uni-versity, Harbin 150040, China

† These authors contributed equally to this work.

* Correspondence: fengwang@nefu.edu.cn (F.W.); Tel: +86-0451-82190384

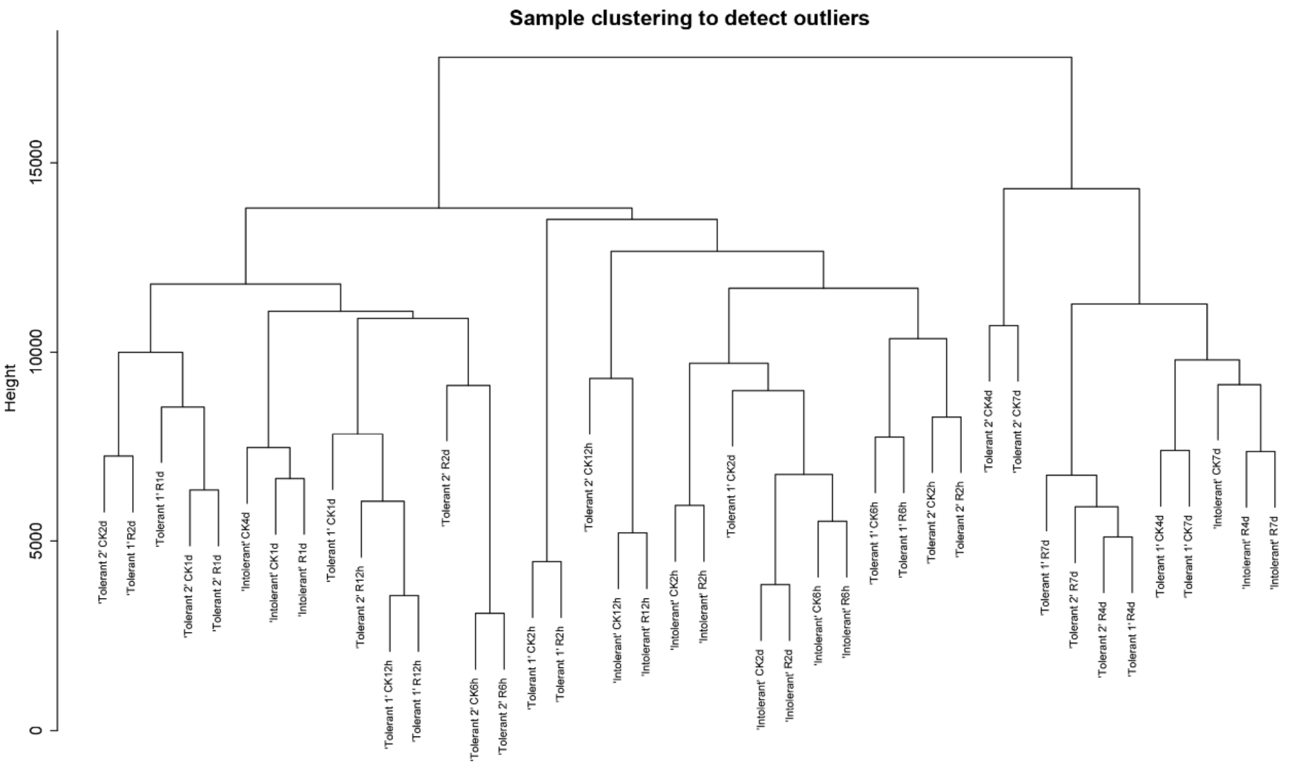

Figure S1. Sample clustering to detect outliers.

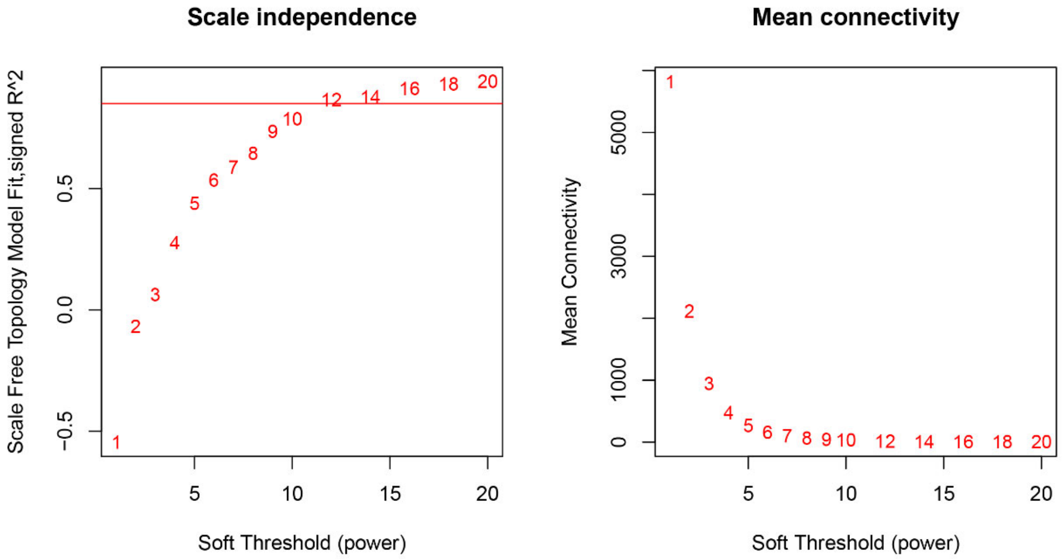

Figure S2. Soft power selection.

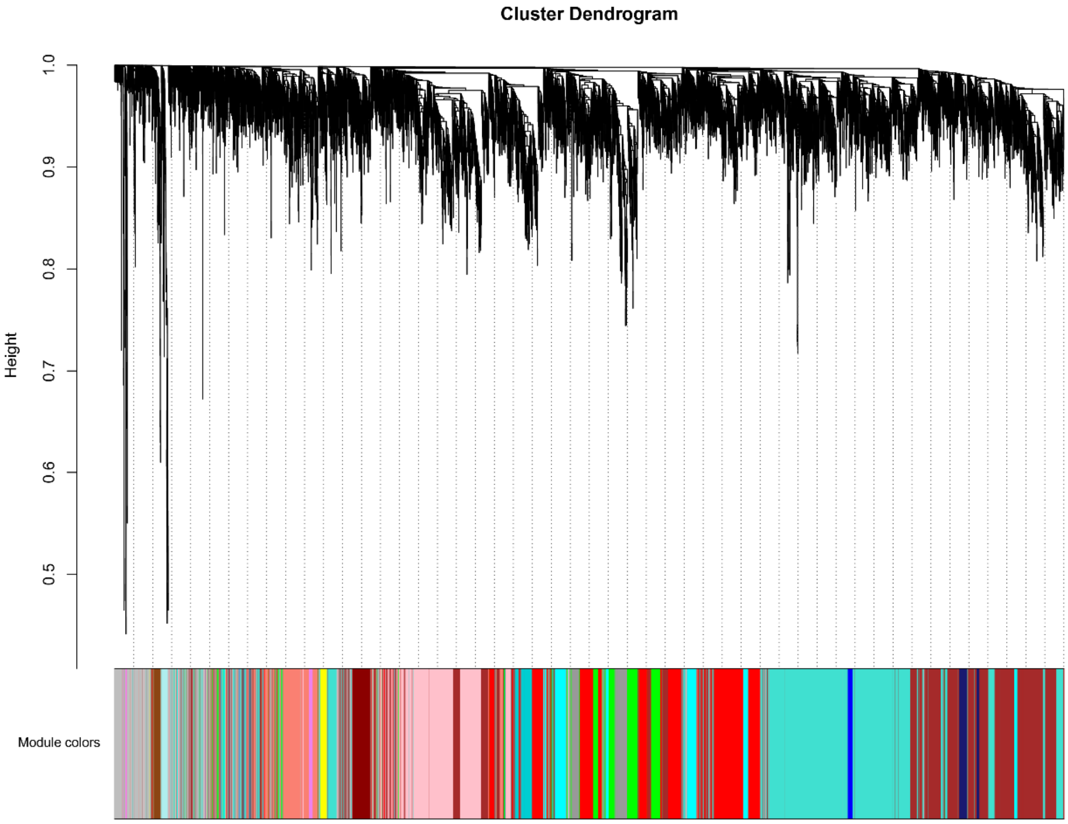

Figure S3. Cluster dendrogram.

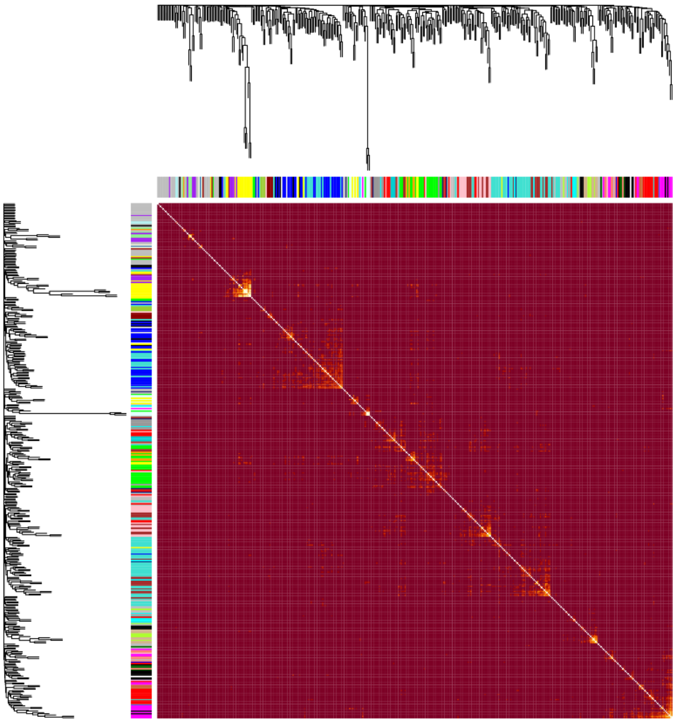

Figure S4. Visualizing the gene network using a heatmap plot (400 genes were randomly selected). The heatmap depicts the Topological Overlap Matrix (TOM) among all genes in the analysis. Dark color represents low overlap and progressively lighter red color represents higher overlap. Blocks of lighter colors along the diagonal are the modules. The gene dendrogram and module assignment are also shown along the left side and the top.

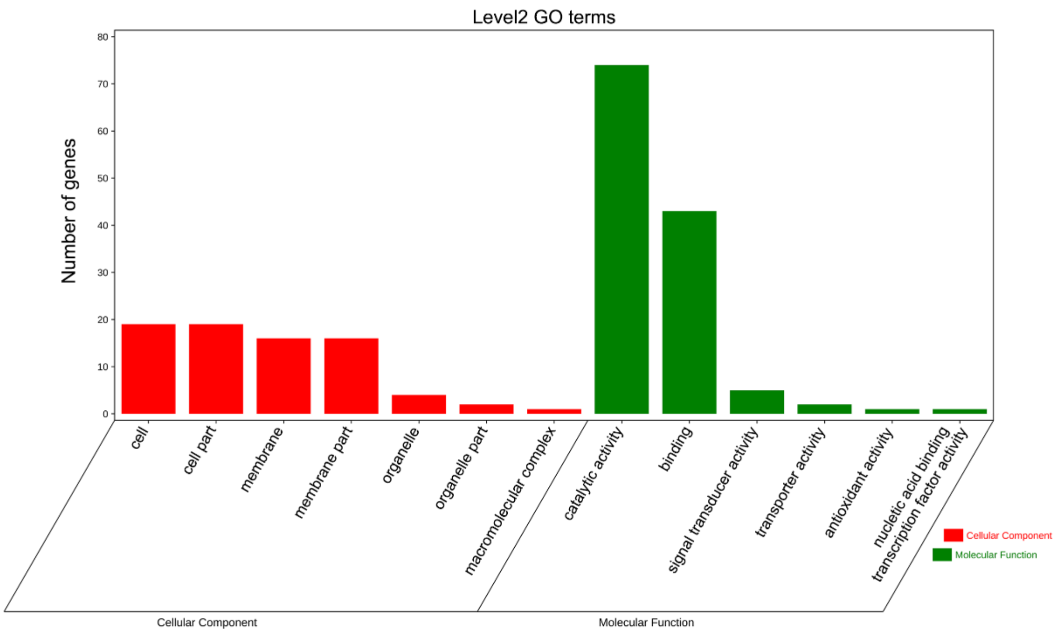

Figure S5. GO enrichment analysis of the JA-related module.

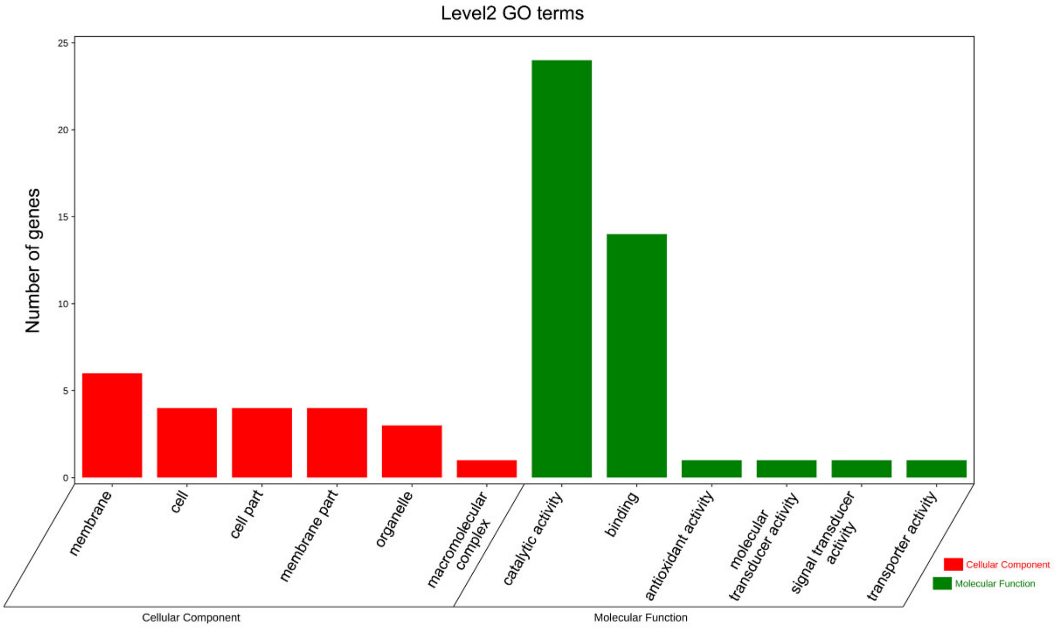

Figure S6. GO enrichment analysis of the bound SA-related module.

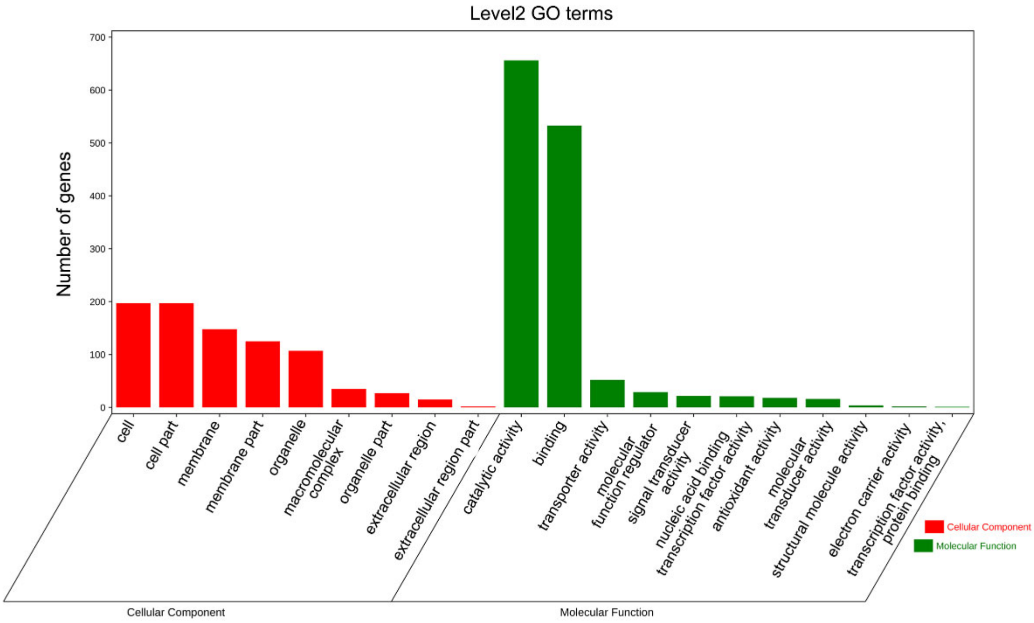

Figure S7. GO enrichment analysis of the free SA-related module.

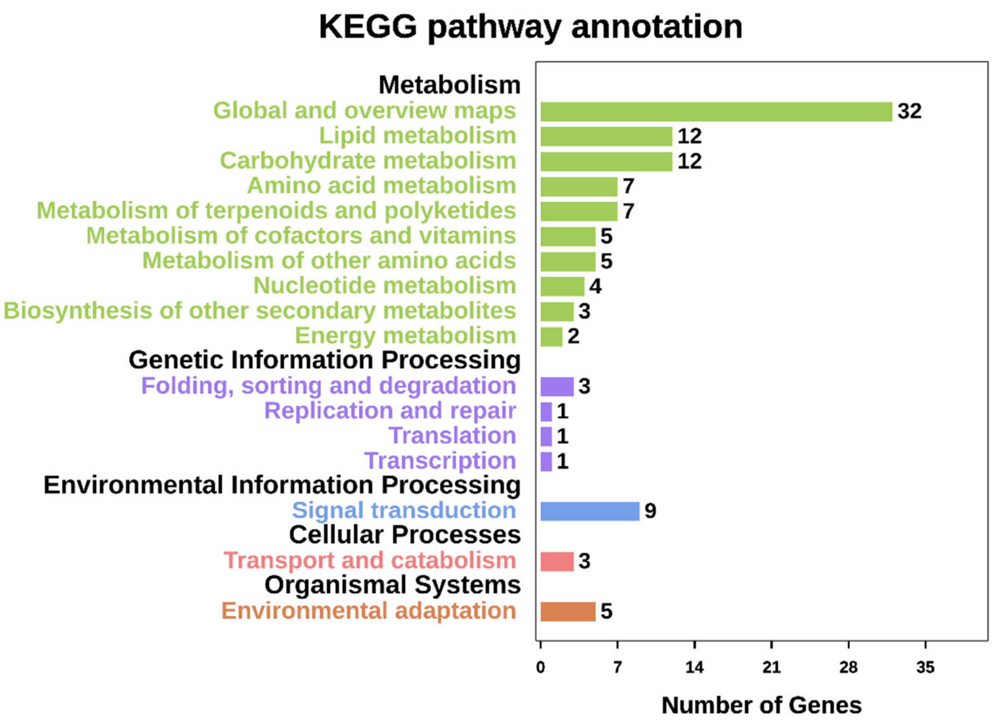

Figure S8. KEGG enrichment analysis of the JA-related module.

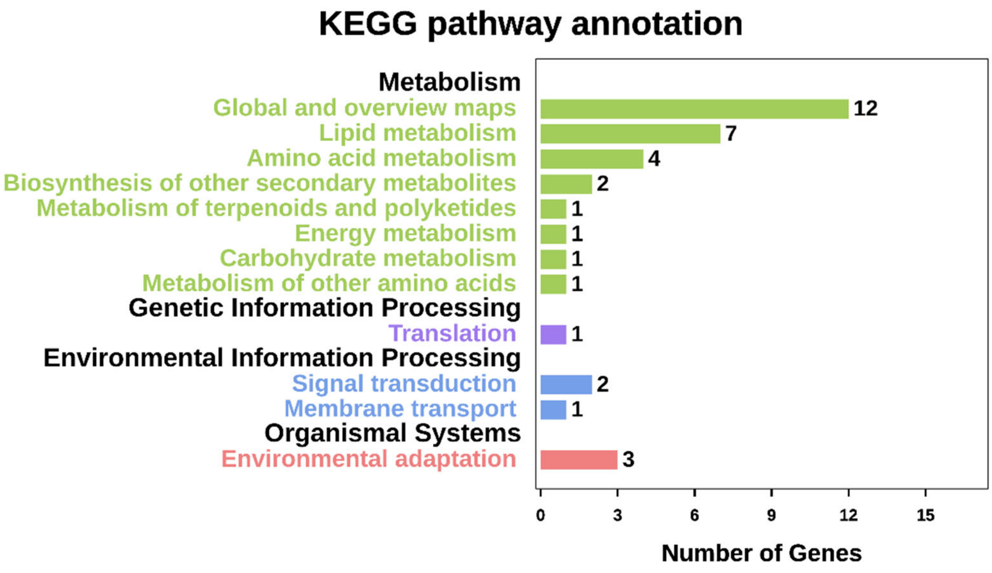

Figure S9. KEGG enrichment analysis of the bound SA-related module.

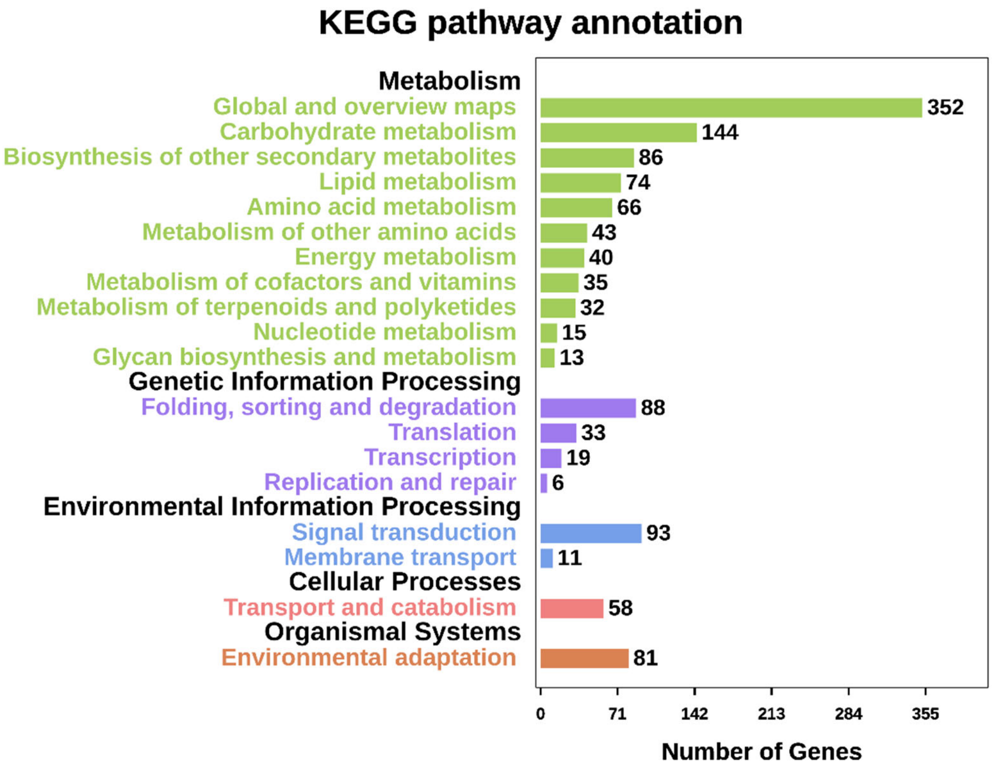

Figure S10. KEGG enrichment analysis of the free SA-related module.

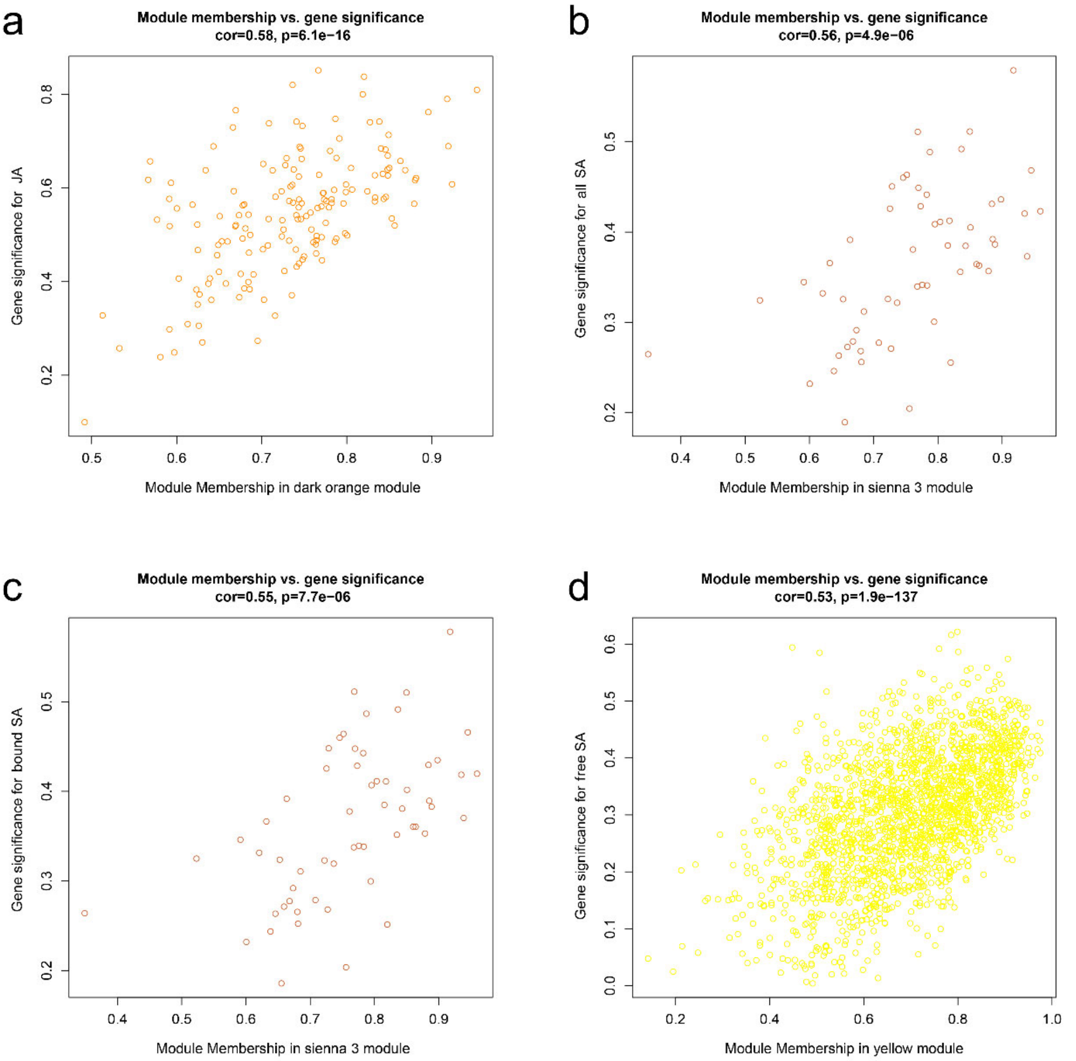

Figure S11. Scatterplot of GS vs. MM in the selected modules. (**a**) GS for JA vs. MM in the JA-related module. (**b**) GS for all SA vs. MM in the bound SA-related module. (**c**) GS for bound SA vs. MM in the bound SA-related module. **(d**) GS for free SA vs. MM in the orange module.

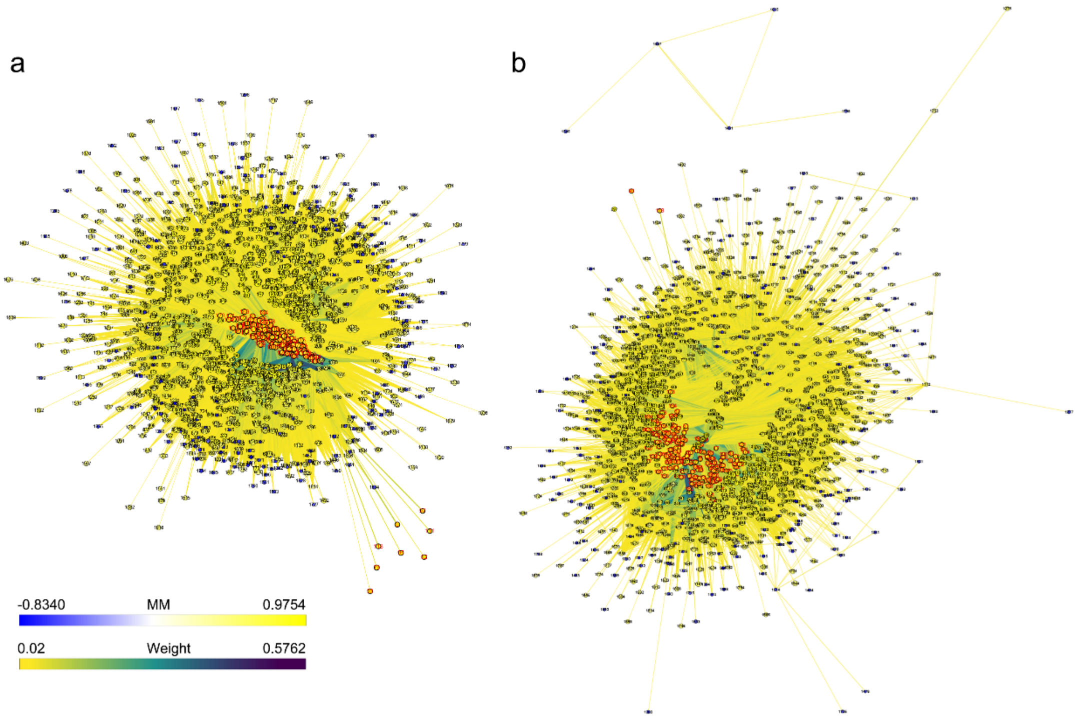

Figure S12. WGCNA revealed gene-network modules highly related to free SA. **(a)** Gene network for genes with the top 10% highest IC values and highly related to free SA. (**b)** Gene network for genes associated with plant resistance and interactions between plants and pathogens and highly related to free SA. The size of the dots represents GS (from -0.5164 to 0.6217). The color of the dots represents MM (from -0.8340 to 0.9754). The colors of the lines represent the weight value between two genes (from 0.0200 to 0.5762). The label of dots is listed based on IC (from 0.0946 to 148.6302), and the hub genes are highlighted with red color labels.

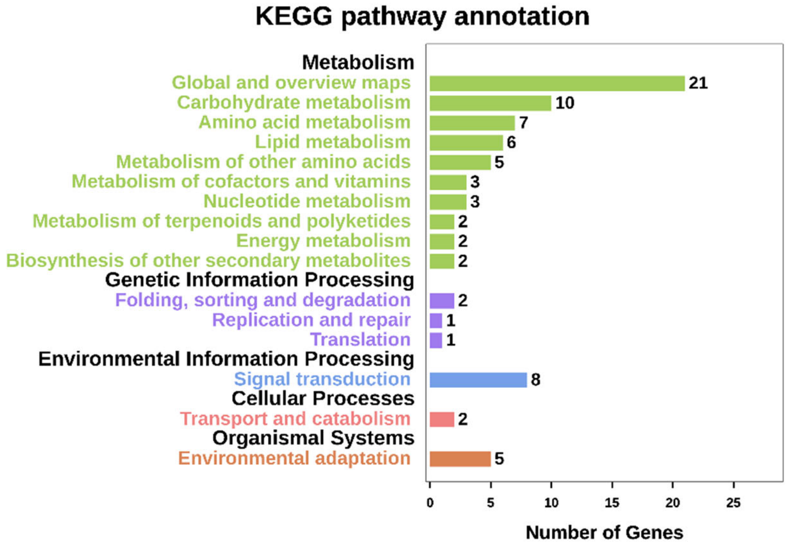

Figure S13. KEGG pathway analysis of the 12 genes enriched in pathways associated with plant defence, and the other 99 genes that these 12 genes might regulate with in the JA-related module.

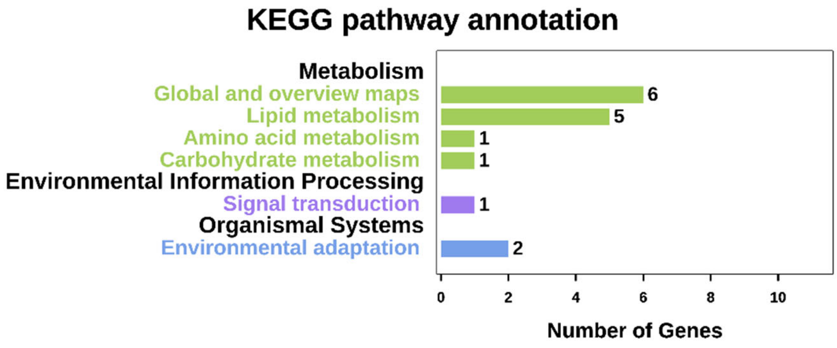

Figure S14. KEGG pathway analysis of the 4 genes enriched in pathways associated with plant defence, and the other 10 genes that these 4 genes might regulate with in the bound SA-related module.

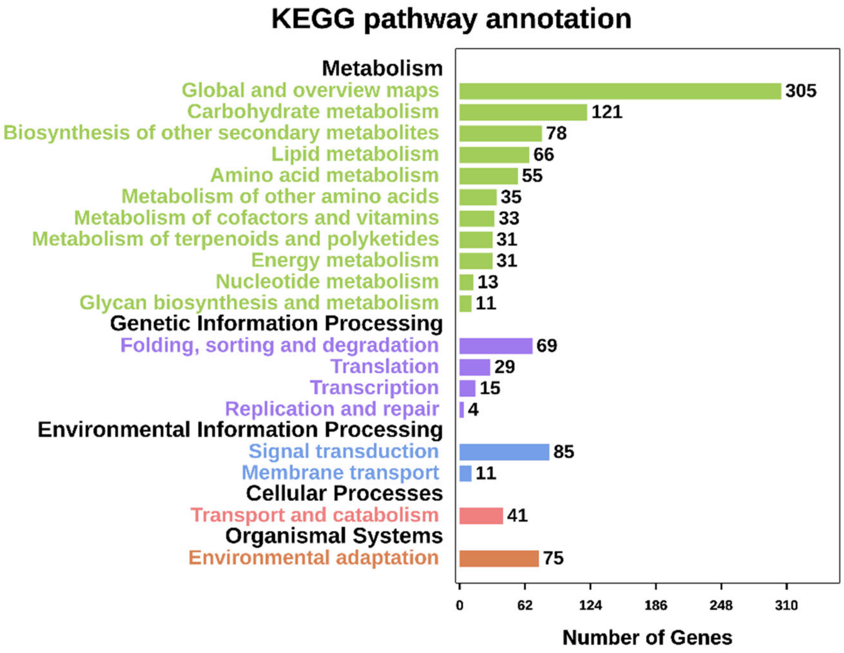

Figure S15. KEGG pathway analysis of the 132 genes enriched in pathways associated with plant defence, and the other 1,757 genes that these 132 genes might regulate with in free SA-related module.

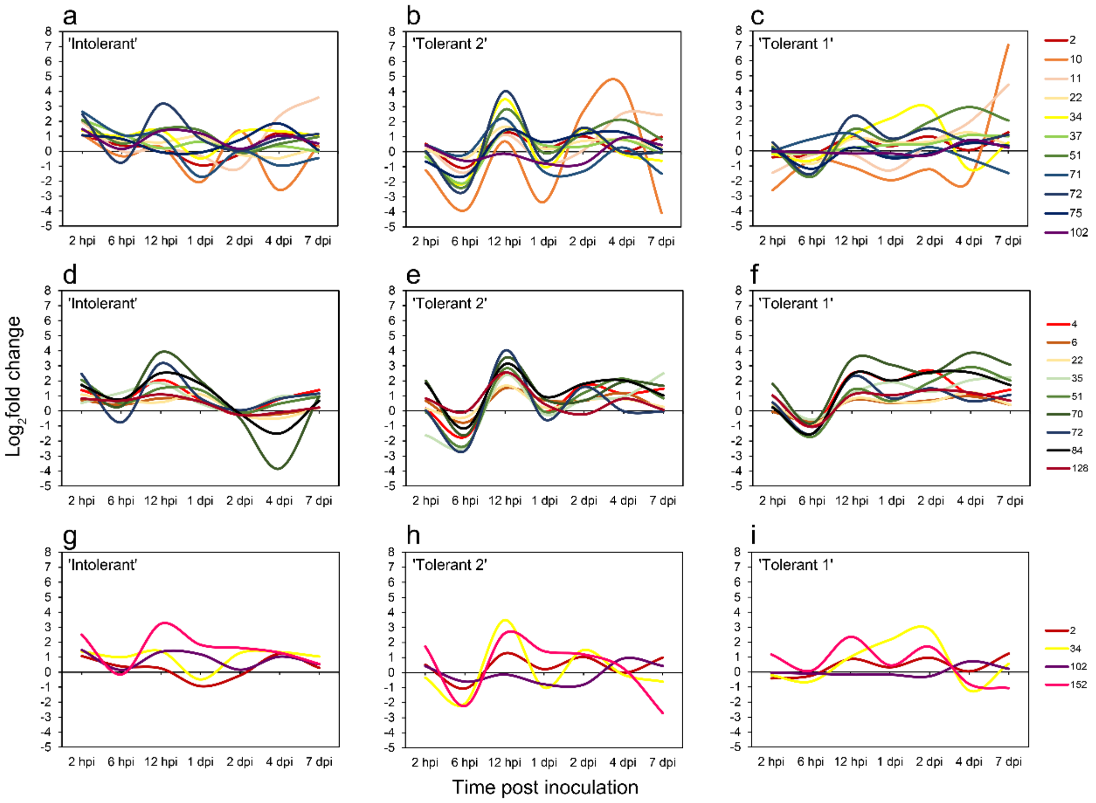

Figure S16. Expression analysis for plant defence-related genes in JA-related module at different time point of E4 infection. (**a, d, g**) Expression analysis of plant defence-related genes in 'Intolerant'. (**b, e, h**) Expression analysis of plant defence-related genes in 'Tolerant 2'. (**c, f, i**) Expression analysis of plant defence-related genes in 'Tolerant 1'.

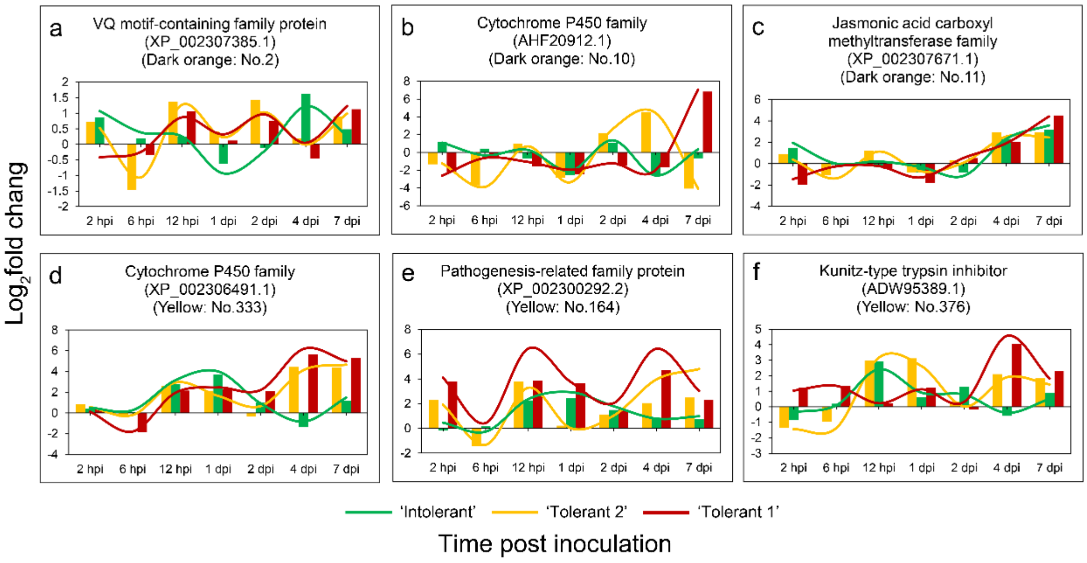

Figure S17. Comparison of the expression changes of selected hub genes among three poplars. Lines represent changes in expression levels based on FPKM and bars represent changes in expression levels verified by RT-qPCR.

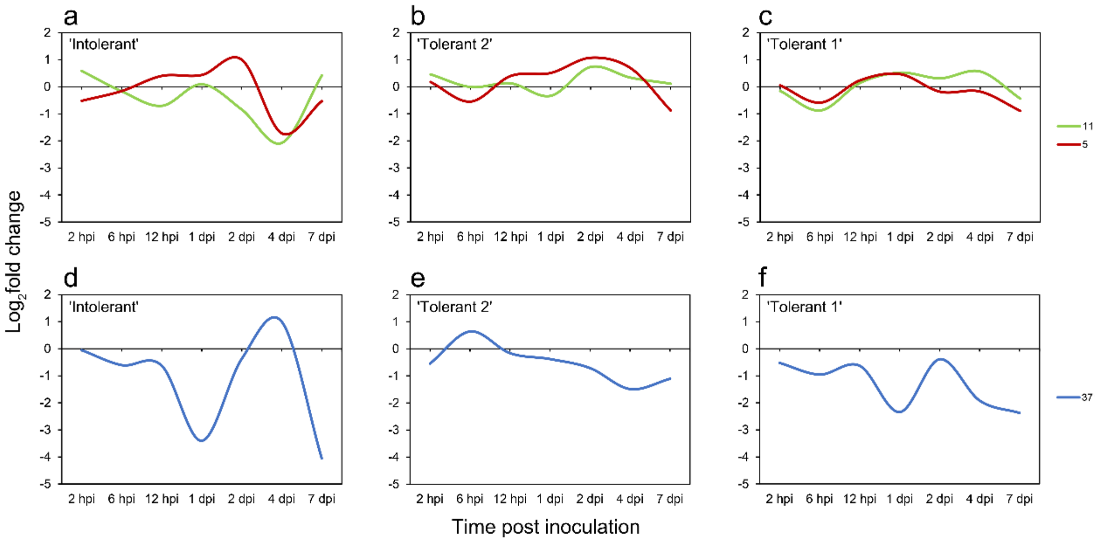

Figure S18. Expression analysis for plant defence-related genes in bound SA-related module at different time point of E4 infection. (**a, d**) Expression analysis of plant defence-related genes in 'Intolerant'. (**b, e**) Expression analysis of plant defence-related genes in 'Tolerant 2'. (**c, f**) Expression analysis of plant defence-related genes in 'Tolerant 1'.

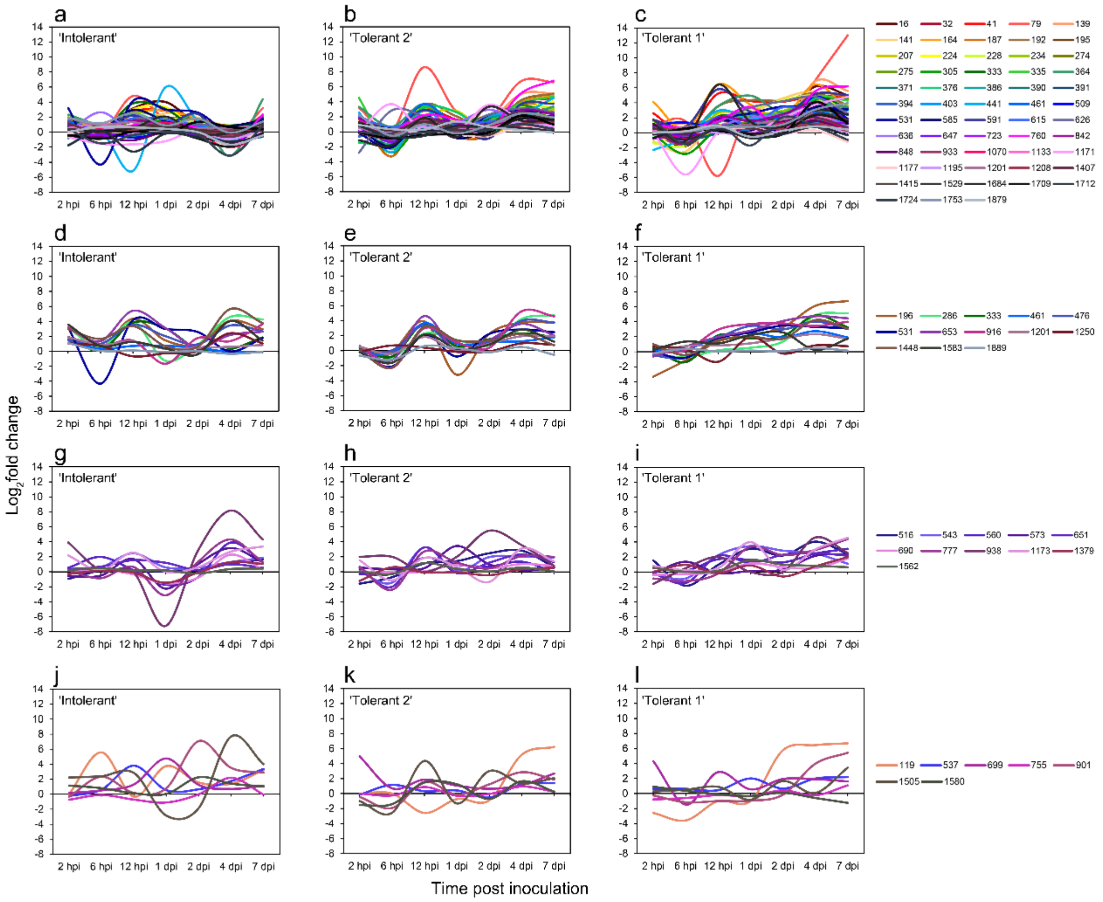

Figure S19. Expression analysis for plant defence-related genes in free SA-related module at different time point of E4 infection. (**a, d, g, j**) Expression analysis of plant defence-related genes in 'Intolerant'. (**b, e, h, k**) Expression analysis of plant defence-related genes in 'Tolerant 2'. (**c, f, i, l**) Expression analysis of plant defence-related genes in 'Tolerant 1'.

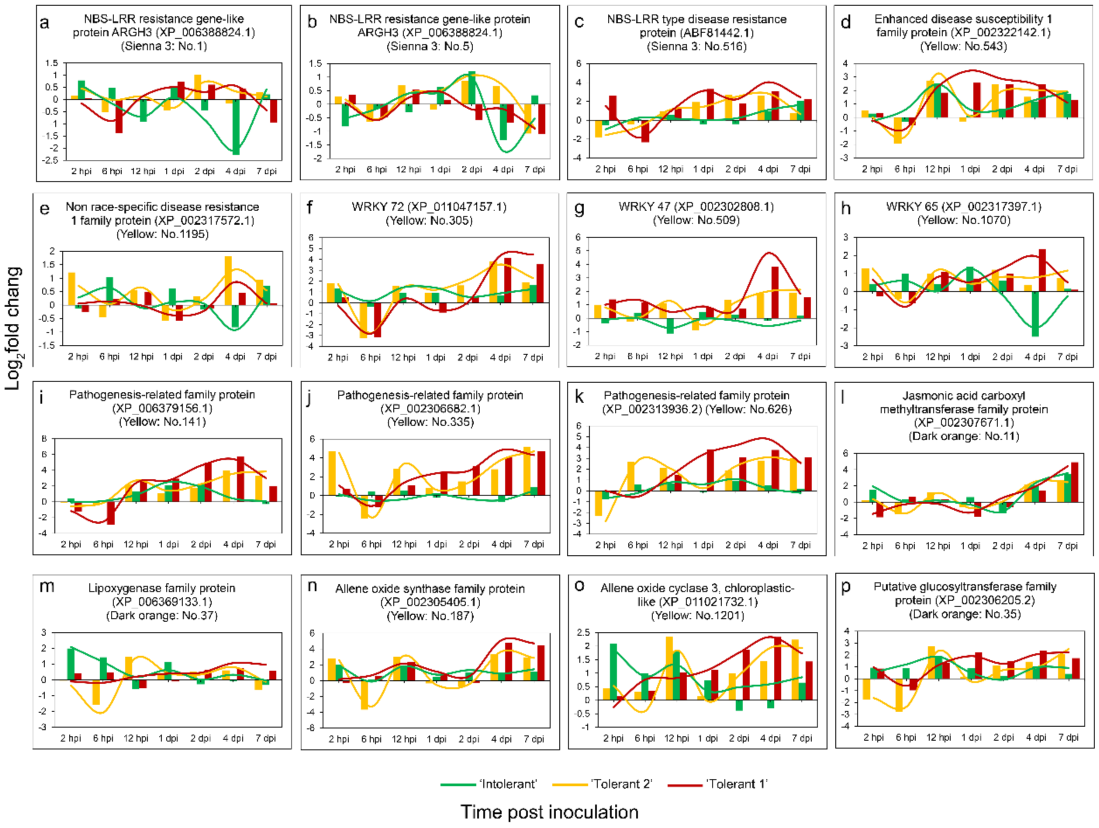

Figure S20. Comparison of the expression changes of selected genes among three poplars. Lines represent changes in expression levels based on FPKM and bars represent changes in expression levels verified by RT-qPCR.

Table S1. Quality statistics of filtered reads.

Sample*	Total raw reads (Mb)	Total clean reads (Mb)	Total clean bases (Gb)	Clean reads Q20 (%)	Clean reads Q30 (%)	Clean reads ratio (%)
'Tolerant 2'CK2h	24.14	23.6	1.18	98.03	89.67	97.78
'Tolerant 2'CK6h	24.14	23.22	1.16	97.88	89.38	96.2
'Tolerant 2'CK12h	26.22	23.54	1.18	98.34	90.57	89.76
'Tolerant 2'CK1d	24.14	22.81	1.14	98.3	90.28	94.51
'Tolerant 2'CK2d	24.14	23.35	1.17	98.4	90.54	96.73
'Tolerant 2'CK4d	24.14	23.35	1.17	97.94	88.6	96.73
'Tolerant 2'CK7d	23.12	20.99	1.05	97.98	90.44	90.79
'Tolerant 2'R2h	24.14	23.42	1.17	98.01	89.58	97.01
'Tolerant 2'R6h	24.14	23.3	1.17	97.82	89.13	96.55
'Tolerant 2'R12h	24.14	22.64	1.13	97.88	89.13	93.79
'Tolerant 2'R1d	24.14	22.11	1.11	98.36	90.44	91.6
'Tolerant 2'R2d	24.14	22.29	1.11	98.27	90.31	92.36
'Tolerant 2'R4d	24.14	22.87	1.14	98.2	90.18	94.76
'Tolerant 2'R7d	24.12	23.82	1.19	98.29	91.19	98.75
'Intolerant' CK2h	26.33	22.66	1.13	97.79	89.31	86.06
'Intolerant' CK6h	26.33	23.9	1.2	98.03	90.04	90.78
'Intolerant' CK12h	24.14	22.98	1.15	98.12	90.07	95.2
'Intolerant' CK1d	24.14	23.66	1.18	97.86	89.18	98.03
'Intolerant' CK2d	26.33	22.89	1.14	98.02	89.99	86.94

‘Intolerant’ CK4d	26.33	23.24	1.16	97.9	89.44	88.25
‘Intolerant’ CK7d	24.14	24.08	1.2	98.56	92.27	99.76
‘Intolerant’ R2h	24.14	23.46	1.17	97.88	89.35	97.21
‘Intolerant’ R6h	24.14	22.52	1.13	97.8	88.9	93.31
‘Intolerant’ R12h	24.14	22.94	1.15	97.98	89.68	95.03
‘Intolerant’ R1d	24.14	23.62	1.18	98.12	90.08	97.88
‘Intolerant’ R2d	26.33	23.66	1.18	97.98	89.78	89.85
‘Intolerant’ R4d	28.53	23.33	1.17	97.48	88.56	81.79
‘Intolerant’ R7d	24.14	23.66	1.18	98.69	92.61	98
‘Tolerant 1’CK2h	24.1	24.04	1.2	98.27	91.07	99.75
‘Tolerant 1’CK6h	23.94	23.8	1.19	98.22	90.91	99.42
‘Tolerant 1’CK12h	23.95	23.71	1.19	98.13	89.99	98.99
‘Tolerant 1’CK1d	23.94	23.46	1.17	98.5	91.55	98.01
‘Tolerant 1’CK2d	23.89	23.61	1.18	98.44	91.42	98.83
‘Tolerant 1’CK4d	24.14	23.51	1.18	98.04	89.9	97.38
‘Tolerant 1’CK7d	24.14	23.55	1.18	97.8	88.86	97.56
‘Tolerant 1’R2h	24.11	24.02	1.2	98.25	90.94	99.62
‘Tolerant 1’R6h	24.03	23.95	1.2	98.13	90.34	99.66
‘Tolerant 1’R12h	23.96	23.79	1.19	98.56	91.78	99.28
‘Tolerant 1’R1d	23.99	23.68	1.18	98.36	90.87	98.72
‘Tolerant 1’R2d	23.9	23.5	1.17	98.6	92.09	98.33
‘Tolerant 1’R4d	23.91	23.31	1.17	98.41	91.28	97.46

‘Tolerant 1’R7d	24.14	23.39	1.17	97.95	89.62	96.88

* CK is short for control check, R is short for rust inoculated, h is short for hour, and d is short for day.

Table S2. Number of genes in each module.

Module	Gene number
black	1148
blue	1952
brown	1900
cyan	432
dark green	227
dark grey	201
dark magenta	59
dark olive green	62
dark orange	162
dark red	233
dark turquoise	226
green	1722
green yellow	650
grey	2375
grey60	313
light cyan	344
light green	284
light yellow	260
magenta	866
midnight blue	356
orange	167
pale turquoise	66
pink	1061
plum1	37
purple	725
red	1557
royal blue	256
saddle brown	89
salmon	548
sienna3	58
sky blue	95
sky blue3	45
steel blue	72
tan	555
turquoise	2819
violet	62
white	125
yellow	1891
yellow green	54

Table S3. Top 20 pathways with the most enriched genes in the JA-related module.

KEGG A class	KEGG B class	Pathway	Count (68 in total)	Pathway ID
Metabolism	Global and overview maps	Metabolic pathways	26	ko01100
Metabolism	Global and overview maps	Biosynthesis of secondary metabolites	25	ko01110
Environmental Information Processing	Signal transduction	Plant hormone signal transduction	6	ko04075
Metabolism	Carbohydrate metabolism	Amino sugar and nucleotide sugar metabolism	5	ko00520
Metabolism	Lipid metabolism	alpha-Linolenic acid metabolism	5	ko00592
Organismal Systems	Environmental adaptation	Plant-pathogen interaction	5	ko04626
Metabolism	Nucleotide metabolism	Purine metabolism	4	ko00230
Metabolism	Global and overview maps	Carbon metabolism	4	ko01200
Environmental Information Processing	Signal transduction	MAPK signaling pathway - plant	4	ko04016
Metabolism	Carbohydrate metabolism	Galactose metabolism	3	ko00052
Metabolism	Amino acid metabolism	Cysteine and methionine metabolism	3	ko00270
Metabolism	Lipid metabolism	Glycerophospholipid metabolism	3	ko00564
Metabolism	Global and overview maps	Biosynthesis of amino acids	3	ko01230
Metabolism	Carbohydrate metabolism	Pentose phosphate pathway	2	ko00030
Metabolism	Carbohydrate metabolism	Fructose and mannose metabolism	2	ko00051
Metabolism	Lipid metabolism	Fatty acid elongation	2	ko00062
Metabolism	Amino acid metabolism	Arginine and proline metabolism	2	ko00330
Metabolism	Amino acid metabolism	Tyrosine metabolism	2	ko00350
Metabolism	Amino acid metabolism	Phenylalanine metabolism	2	ko00360
Metabolism	Metabolism of other amino acids	beta-Alanine metabolism	2	ko00410

Table S4. Top 20 pathways with the most enriched genes in the bound SA-related module.

KEGG A class	KEGG B class	Pathway	Count (19 in total)	Pathway ID
Metabolism	Global and overview maps	Metabolic pathways	11	ko01100
Metabolism	Lipid metabolism	Ether lipid metabolism	3	ko00565
Metabolism	Lipid metabolism	Sphingolipid metabolism	3	ko00600
Metabolism	Global and overview maps	Biosynthesis of secondary metabolites	3	ko01110
Metabolism	Amino acid metabolism	Tryptophan metabolism	2	ko00380
Organismal Systems	Environmental adaptation	Plant-pathogen interaction	2	ko04626
Metabolism	Lipid metabolism	Cutin, suberine and wax biosynthesis	1	ko00073
Metabolism	Lipid metabolism	Steroid biosynthesis	1	ko00100
Metabolism	Biosynthesis of other secondary metabolites	Caffeine metabolism	1	ko00232
Metabolism	Amino acid metabolism	Cysteine and methionine metabolism	1	ko00270
Metabolism	Amino acid metabolism	Arginine and proline metabolism	1	ko00330
Metabolism	Metabolism of other amino acids	beta-Alanine metabolism	1	ko00410
Metabolism	Lipid metabolism	Glycerolipid metabolism	1	ko00561
Metabolism	Lipid metabolism	Linoleic acid metabolism	1	ko00591
Metabolism	Carbohydrate metabolism	Glyoxylate and dicarboxylate metabolism	1	ko00630
Metabolism	Carbohydrate metabolism	Butanoate metabolism	1	ko00650
Metabolism	Biosynthesis of other secondary metabolites	Indole alkaloid biosynthesis	1	ko00901
Metabolism	Metabolism of terpenoids and polyketides	Sesquiterpenoid and triterpenoid biosynthesis	1	ko00909
Metabolism	Energy metabolism	Sulfur metabolism	1	ko00920
Metabolism	Global and overview maps	Carbon metabolism	1	ko01200

Table S5. Top 20 pathways with the most enriched genes in the free SA-related module.

KEGG A class	KEGG B class	Pathway	Count (735 in total)	Pathway ID
Metabolism	Global and overview maps	Metabolic pathways	325	ko01100
Metabolism	Global and overview maps	Biosynthesis of secondary metabolites	191	ko01110
Organismal Systems	Environmental adaptation	Plant-pathogen interaction	76	ko04626
Environmental Information Processing	Signal transduction	MAPK signaling pathway - plant	55	ko04016
Metabolism	Biosynthesis of other secondary metabolites	Phenylpropanoid biosynthesis	49	ko00940
Genetic Information Processing	Folding, sorting and degradation	Protein processing in endoplasmic reticulum	48	ko04141
Environmental Information Processing	Signal transduction	Plant hormone signal transduction	46	ko04075
Metabolism	Global and overview maps	Carbon metabolism	41	ko01200
Metabolism	Carbohydrate metabolism	Starch and sucrose metabolism	38	ko00500
Metabolism	Global and overview maps	Biosynthesis of amino acids	33	ko01230
Metabolism	Carbohydrate metabolism	Amino sugar and nucleotide sugar metabolism	32	ko00520
Cellular Processes	Transport and catabolism	Endocytosis	32	ko04144
Metabolism	Metabolism of other amino acids	Glutathione metabolism	22	ko00480
Metabolism	Amino acid metabolism	Cysteine and methionine metabolism	18	ko00270
Metabolism	Metabolism of other amino acids	Cyanoamino acid metabolism	17	ko00460
Genetic Information Processing	Folding, sorting and degradation	RNA degradation	17	ko03018
Metabolism	Lipid metabolism	Glycerophospholipid metabolism	15	ko00564
Metabolism	Biosynthesis of other secondary metabolites	Flavonoid biosynthesis	15	ko00941
Cellular Processes	Transport and catabolism	Phagosome	15	ko04145
Metabolism	Carbohydrate metabolism	Glycolysis / Gluconeogenesis	14	ko00010

Table S6. Pathways associated with plant defence.

	KEGG A class	KEGG B class	Pathway	Gene amount	Pathway ID
JA-related	Environmental Information Processing	Signal transduction	MAPK signaling pathway - plant	4	ko04016
	Environmental Information Processing	Signal transduction	Plant hormone signal transduction	6	ko04075
	Organismal Systems	Environmental adaptation	Plant-pathogen interaction	5	ko04626
bound SA-related	Organismal Systems	Environmental adaptation	Plant-pathogen interaction	2	ko04626
	Metabolism	Lipid metabolism	Cutin, suberine and wax biosynthesis	1	ko00073
	Environmental Information Processing	Signal transduction	MAPK signaling pathway - plant	1	ko04016
	Environmental Information Processing	Signal transduction	Plant hormone signal transduction	1	ko04075
Free SA-related	Environmental Information Processing	Signal transduction	MAPK signaling pathway - plant	55	ko04016
	Environmental Information Processing	Signal transduction	Plant hormone signal transduction	46	ko04075
	Organismal Systems	Environmental adaptation	Plant-pathogen interaction	76	ko04626

Table S7. Genes with the top 10% highest IC values in the JA-related module.

No.	IC value	Homologous protein ID	NR description
1	5.973528566	XP_002314709.1	hypothetical protein POPTR_0010s10010g (*Populus trichocarpa*)
2	4.864668966	XP_002307385.1	VQ motif-containing family protein (*Populus trichocarpa*)
3	4.691983745	XP_011045724.1	PREDICTED: bifunctional riboflavin biosynthesis protein RIBA 1, chloroplastic (*Populus euphratica*)
4	4.548884762	XP_002321141.2	LysM domain-containing receptor-like kinase 4 family protein (*Populus trichocarpa*)
5	4.446890778	XP_002306245.2	hypothetical protein POPTR_0005s06380g (*Populus trichocarpa*)
6	4.164548353	XP_002324557.1	calmodulin-binding protein 60-D (*Populus trichocarpa*)
7	4.147102242	XP_002314201.1	nodulin-26 family protein (*Populus trichocarpa*)
8	3.924833276	XP_002325025.1	hypothetical protein POPTR_0018s09440g (*Populus trichocarpa*)
9	3.765921097	XP_002309511.2	hypothetical protein POPTR_0006s24820g (*Populus trichocarpa*)
10	3.589766438	AHF20912.1	cytochrome P450 (*Populus trichocarpa*)
11	3.547475821	XP_002307671.1	JASMONIC ACID CARBOXYL METHYLTRANSFERASE family protein (*Populus trichocarpa*)
12	3.417102322	XP_002312868.2	hypothetical protein POPTR_0009s15650g (*Populus trichocarpa*)
13	3.177433969	XP_002322937.1	hypothetical protein POPTR_0016s09980g (*Populus trichocarpa*)
14	3.159653387	XP_002306141.1	arginine decarboxylase family protein (*Populus trichocarpa*)

15	3.058834128	XP_006379054.1	hypothetical protein POPTR_0009s05300g (*Populus trichocarpa*)
16	3.039848584	XP_002312869.2	hypothetical protein POPTR_0009s15610g (*Populus trichocarpa*)

Table S8. Genes with the top 30 highest IC values in the bound SA-related module.

No.	IC value	Homologous protein ID	NR description
1	3.943590186	XP_006388824.1	NBS-LRR resistance gene-like protein ARGH30 (*Populus trichocarpa*)
2	3.309735602	XP_006372437.1	hypothetical protein POPTR_0017s01630g (*Populus trichocarpa*)
3	3.189142657	XP_006372431.1	hypothetical protein POPTR_0017s01570g (*Populus trichocarpa*)
4	2.941401632	XP_011008563.1	PREDICTED: uncharacterized protein LOC105113904 (*Populus euphratica*)
5	2.79383339	XP_006388824.1	NBS-LRR resistance gene-like protein ARGH30 (*Populus trichocarpa*)
6	2.778783243	XP_006373375.1	hypothetical protein POPTR_0017s13190g, partial (*Populus trichocarpa*)

Table S9. Genes with the top 30 highest IC values in the free SA-related module.

No.	IC value	Homologous protein ID	NR description
1	148.6302142	XP_002323320.2	Heat Stress Transcription Factor family protein (*Populus trichocarpa*)
2	138.1544771	XP_002306323.2	L-ascorbate oxidase family protein (*Populus trichocarpa*)
3	136.3722566	XP_006374448.1	leucine-rich repeat transmembrane protein kinase (*Populus trichocarpa*)
4	134.8485406	XP_002314892.1	hypothetical protein POPTR_0010s14140g (*Populus trichocarpa*)
5	134.1160765	XP_011002676.1	PREDICTED: E3 ubiquitin-protein ligase ATL41-like (*Populus euphratica*)
6	134.0584355	XP_002319067.1	MtN26 family protein (*Populus trichocarpa*)
7	133.7729057	XP_002317208.1	pectin methylesterase family protein (*Populus trichocarpa*)
8	133.3746685	XP_002300139.1	hypothetical protein POPTR_0001s33050g (*Populus trichocarpa*)
9	132.4188616	XP_006385099.1	hypothetical protein POPTR_0004s23910g (*Populus trichocarpa*)
10	132.1581919	XP_011048077.1	PREDICTED: heat stress transcription factor B-3 (*Populus euphratica*)
11	132.1360659	XP_011022380.1	PREDICTED: transcription factor JUNGBRUNNEN 1-like (*Populus euphratica*)
12	130.7577068	XP_002303031.1	hypothetical protein POPTR_0002s24200g (*Populus trichocarpa*)
13	129.6874685	XP_006381896.1	hypothetical protein POPTR_0006s20270g (*Populus trichocarpa*)
14	129.2553016	XP_002298154.1	cysteine protease inhibitor family protein (*Populus trichocarpa*)
15	126.4754151	XP_006380217.1	hypothetical protein POPTR_0008s23030g (*Populus trichocarpa*)
16	125.9588436	XP_002306491.1	cytochrome P450 family protein (*Populus trichocarpa*)
17	124.8273619	XP_006386464.1	hypothetical protein POPTR_0002s11520g (*Populus trichocarpa*)
18	124.4024963	XP_002317074.2	FAD-binding domain-containing family protein (*Populus trichocarpa*)
19	124.1402552	XP_002309886.1	U-box domain-containing family protein (*Populus trichocarpa*)
20	123.968809	XP_002322663.2	hypothetical protein POPTR_0016s04410g (*Populus trichocarpa*)
21	123.6021274	XP_006376177.1	hypothetical protein POPTR_0013s10540g (*Populus trichocarpa*)
22	123.3207905	XP_002309883.1	hypothetical protein POPTR_0007s03640g (*Populus trichocarpa*)
23	123.0524033	XP_002299232.1	hypothetical protein POPTR_0001s05690g (*Populus*

			trichocarpa)
24	122.863476	XP_002307288.2	hypothetical protein POPTR_0005s18660g (*Populus trichocarpa*)
25	122.7758249	XP_002320148.2	hypothetical protein POPTR_0014s08350g (*Populus trichocarpa*)
26	120.9562242	XP_006378351.1	hypothetical protein POPTR_0010s08590g (*Populus trichocarpa*)
27	120.9509817	XP_002309424.2	hypothetical protein POPTR_0006s22860g (*Populus trichocarpa*)
28	120.8475033	XP_006383679.1	hypothetical protein POPTR_0005s23720g (*Populus trichocarpa*)
29	119.1449245	XP_002300413.2	hypothetical protein POPTR_0001s38380g (*Populus trichocarpa*)
30	118.7721645	XP_002313569.2	hypothetical protein POPTR_0009s17090g (*Populus trichocarpa*)
31	118.6608475	XP_002316320.1	hydrolase family protein (*Populus trichocarpa*)
32	118.2384071	XP_002324257.1	K+ rectifying channel family protein (*Populus trichocarpa*)
33	118.2196685	XP_011004115.1	PREDICTED: inhibitor of trypsin and hageman factor-like (*Populus euphratica*)
34	117.9576904	XP_006381983.1	hypothetical protein POPTR_0006s22880g (*Populus trichocarpa*)
35	116.7260168	XP_006370472.1	hypothetical protein POPTR_0001s43030g (*Populus trichocarpa*)
36	116.7258855	XP_002308364.2	hypothetical protein POPTR_0006s20740g (*Populus trichocarpa*)
37	116.7232045	XP_002316348.2	hypothetical protein POPTR_0010s22550g (*Populus trichocarpa*)
38	116.4229559	XP_006386332.1	hypothetical protein POPTR_0002s07480g (*Populus trichocarpa*)
39	115.704577	XP_006387619.1	hypothetical protein POPTR_0770s00220g (*Populus trichocarpa*)
40	115.262614	XP_002303937.1	hypothetical protein POPTR_0003s19420g (*Populus trichocarpa*)
41	114.516588	XP_011007351.1	PREDICTED: cytochrome P450 87A3-like (*Populus euphratica*)
42	114.3789903	XP_002306399.2	hypothetical protein POPTR_0005s09830g (*Populus trichocarpa*)
43	114.2433155	XP_006381260.1	hypothetical protein POPTR_0006s11180g (*Populus trichocarpa*)
44	114.1431321	XP_002311468.2	hypothetical protein POPTR_0008s12260g (*Populus trichocarpa*)
45	114.0514493	XP_002310332.2	hypothetical protein POPTR_0007s14820g (*Populus trichocarpa*)
46	113.5799054	XP_006387620.1	hypothetical protein POPTR_0770s00230g (*Populus

			trichocarpa)
47	113.0863196	XP_011006155.1	PREDICTED: probable glucan 1,3-beta-glucosidase A (*Populus euphratica*)
48	112.5164408	XP_002323542.2	hypothetical protein POPTR_0016s11600g (*Populus trichocarpa*)
49	111.922694	AES12473.1	C2H2-type zinc finger protein 1 (*Populus trichocarpa*)
50	111.5614647	XP_006375299.1	hypothetical protein POPTR_0014s06050g (*Populus trichocarpa*)
51	111.3127306	XP_002316906.2	hypothetical protein POPTR_0011s12180g (*Populus trichocarpa*)
52	111.1065251	XP_002314939.2	hypothetical protein POPTR_0010s15320g (*Populus trichocarpa*)
53	110.716015	XP_006384235.1	hypothetical protein POPTR_0004s10900g (*Populus trichocarpa*)
54	110.595504	XP_002323865.1	hypothetical protein POPTR_0017s12100g (*Populus trichocarpa*)
55	110.5334727	XP_011037985.1	PREDICTED: uncharacterized protein LOC105135011 (*Populus euphratica*)
56	110.4890797	XP_006384880.1	hypothetical protein POPTR_0004s21910g (*Populus trichocarpa*)
57	110.1107081	XP_002316607.1	hypothetical protein POPTR_0011s00410g (*Populus trichocarpa*)
58	110.0716232	XP_002299656.2	hypothetical protein POPTR_0001s22170g (*Populus trichocarpa*)
59	110.0274643	XP_002300267.1	hypothetical protein POPTR_0001s30200g (*Populus trichocarpa*)
60	110.0155961	XP_002318799.2	NDR1/HIN1-LIKE 25 family protein (*Populus trichocarpa*)
61	110.011132	XP_002314934.2	Glucan endo-1 family protein (*Populus trichocarpa*)
62	109.7356659	XP_011029500.1	PREDICTED: uncharacterized protein LOC105129221 (*Populus euphratica*)
63	109.4627459	XP_002317084.1	hypothetical protein POPTR_0011s16140g (*Populus trichocarpa*)
64	108.9600744	XP_006373298.1	hypothetical protein POPTR_0017s11230g (*Populus trichocarpa*)
65	107.9451984	XP_002312093.2	hypothetical protein POPTR_0008s05530g (*Populus trichocarpa*)
66	107.5111805	XP_006377946.1	hypothetical protein POPTR_0011s16570g (*Populus trichocarpa*)
67	107.1898208	XP_002305935.2	hypothetical protein POPTR_0004s07200g (*Populus trichocarpa*)
68	106.644026	XP_002300412.2	hypothetical protein POPTR_0001s38370g (*Populus trichocarpa*)
69	106.5182663	XP_006370473.1	hypothetical protein POPTR_0001s43040g (*Populus*

			trichocarpa)
70	106.4352962	XP_002316840.2	hypothetical protein POPTR_0011s07770g (*Populus trichocarpa*)
71	105.6960765	XP_002301550.2	hypothetical protein POPTR_0002s18850g (*Populus trichocarpa*)
72	105.303047	XP_002317087.1	hypothetical protein POPTR_0011s16180g (*Populus trichocarpa*)
73	104.8060315	XP_006371753.1	hypothetical protein POPTR_0018s01920g (*Populus trichocarpa*)
74	104.8017431	XP_002314644.1	hypothetical protein POPTR_0010s08610g (*Populus trichocarpa*)
75	104.6054478	XP_002323045.1	hypothetical protein POPTR_0016s13890g (*Populus trichocarpa*)
76	104.3070516	XP_002305253.2	hypothetical protein POPTR_0004s07460g (*Populus trichocarpa*)
77	103.5600716	XP_006384087.1	hypothetical protein POPTR_0004s06660g (*Populus trichocarpa*)
78	103.4924817	XP_011037985.1	PREDICTED: uncharacterized protein LOC105135011 (*Populus euphratica*)
79	103.0886726	ADW95387.1	Kunitz-type trypsin inhibitor (*Populus nigra*)
80	103.0564442	XP_002300513.1	eugenol O-methyltransferase family protein (*Populus trichocarpa*)
81	102.7410435	XP_006375302.1	hypothetical protein POPTR_0014s06100g (*Populus trichocarpa*)
82	102.3024275	XP_002300414.2	hypothetical protein POPTR_0001s38400g (*Populus trichocarpa*)
83	101.9711527	XP_002303483.2	hypothetical protein POPTR_0003s10530g (*Populus trichocarpa*)
84	101.4520349	XP_002310757.2	Chain A family protein (*Populus trichocarpa*)
85	101.1469523	XP_002298258.1	hypothetical protein POPTR_0001s19500g (*Populus trichocarpa*)
86	100.8214529	XP_006371068.1	hypothetical protein POPTR_0019s03190g (*Populus trichocarpa*)
87	100.3327969	XP_006378442.1	hypothetical protein POPTR_0010s11930g (*Populus trichocarpa*)
88	100.0060982	XP_002315654.1	hypothetical protein POPTR_0010s05940g (*Populus trichocarpa*)
89	99.9430928	XP_002316697.1	hypothetical protein POPTR_0011s03570g (*Populus trichocarpa*)
90	99.69800369	XP_002305871.1	hypothetical protein POPTR_0004s08660g (*Populus trichocarpa*)
91	99.49115707	XP_006374943.1	alpha amylase family protein (*Populus trichocarpa*)
92	99.2999701	XP_011046086.1	PREDICTED: GEM-like protein 7 isoform X2 (*Populus euphratica*)

93	99.1749001	XP_006387602.1	hypothetical protein POPTR_0790s00210g (*Populus trichocarpa*)
94	99.13782304	XP_002320916.2	hypothetical protein POPTR_0014s10460g, partial (*Populus trichocarpa*)
95	98.99266953	XP_006371645.1	hypothetical protein POPTR_0019s14090g (*Populus trichocarpa*)
96	98.79004537	XP_002299521.2	hypothetical protein POPTR_0001s09570g (*Populus trichocarpa*)
97	98.63894357	XP_006384235.1	hypothetical protein POPTR_0004s10900g (*Populus trichocarpa*)
98	98.44047424	XP_002301198.2	hypothetical protein POPTR_0002s13000g (*Populus trichocarpa*)
99	97.40850244	XP_002320018.2	hypothetical protein POPTR_0014s01340g (*Populus trichocarpa*)
100	97.3472536	XP_002311193.1	hypothetical protein POPTR_0008s06200g (*Populus trichocarpa*)
101	97.30560019	XP_002318964.2	hypothetical protein POPTR_0013s01200g (*Populus trichocarpa*)
102	97.18607833	XP_006375405.1	hypothetical protein POPTR_0014s10450g (*Populus trichocarpa*)
103	97.16856388	XP_006388087.1	hypothetical protein POPTR_0347s00200g (*Populus trichocarpa*)
104	97.06354967	XP_006378352.1	hypothetical protein POPTR_0010s08600g (*Populus trichocarpa*)
105	96.87487472	XP_002299029.2	hypothetical protein POPTR_0001s46700g (*Populus trichocarpa*)
106	96.75546697	XP_002300163.2	hypothetical protein POPTR_0001s32430g, partial (*Populus trichocarpa*)
107	96.37053064	XP_002320043.1	hypothetical protein POPTR_0014s06090g (*Populus trichocarpa*)
108	96.16781528	XP_002299955.2	hypothetical protein POPTR_0001s27750g (*Populus trichocarpa*)
109	95.7315208	XP_011037985.1	PREDICTED: uncharacterized protein LOC105135011 (*Populus euphratica*)
110	94.84128081	XP_002317460.1	hypothetical protein POPTR_0011s11140g (*Populus trichocarpa*)
111	94.29254484	XP_006371663.1	hypothetical protein POPTR_0019s14550g (*Populus trichocarpa*)
112	93.85837271	XP_002323382.1	U-box domain-containing family protein (*Populus trichocarpa*)
113	93.82902948	XP_002307434.1	early nodulin 8 precursor family protein (*Populus trichocarpa*)
114	93.66027877	XP_006373205.1	hypothetical protein POPTR_0017s09640g (*Populus trichocarpa*)

115	92.70151525	XP_002320355.1	hypothetical protein POPTR_0014s12700g (*Populus trichocarpa*)
116	92.4367527	XP_002299020.2	hypothetical protein POPTR_0001s46600g (*Populus trichocarpa*)
117	91.99684444	XP_002318631.2	hypothetical protein POPTR_0012s07750g (*Populus trichocarpa*)
118	91.99540428	XP_006373216.1	hypothetical protein POPTR_0017s09750g (*Populus trichocarpa*)
119	91.33632844	ADW95385.1	Kunitz-type trypsin inhibitor (*Populus nigra*)
120	91.2431382	XP_002314900.2	hypothetical protein POPTR_0010s14270g (*Populus trichocarpa*)
121	90.92023632	XP_002303764.2	hypothetical protein POPTR_0003s16450g, partial (*Populus trichocarpa*)
122	90.80496325	XP_006368315.1	hypothetical protein POPTR_0001s01540g (*Populus trichocarpa*)
123	90.733723	XP_002310351.1	hypothetical protein POPTR_0007s15210g (*Populus trichocarpa*)
124	90.49180316	XP_002299142.2	hypothetical protein POPTR_0001s04840g (*Populus trichocarpa*)
125	89.73450252	XP_002299545.2	hypothetical protein POPTR_0001s09110g (*Populus trichocarpa*)
126	89.69054934	XP_006377134.1	hypothetical protein POPTR_0011s00400g (*Populus trichocarpa*)
127	89.38843266	XP_002299958.1	hypothetical protein POPTR_0001s27780g (*Populus trichocarpa*)
128	89.36865036	XP_002300411.2	hypothetical protein POPTR_0001s38360g (*Populus trichocarpa*)
129	89.34491574	XP_011013507.1	PREDICTED: epidermis-specific secreted glycoprotein EP1-like (*Populus euphratica*)
130	89.08536329	XP_006383817.1	truncated Kunitz trypsin inhibitor family protein (*Populus trichocarpa*)
131	89.00553231	XP_011043686.1	PREDICTED: ATP-dependent zinc metalloprotease YME1 homolog (*Populus euphratica*)
132	88.78077766	XP_002303758.1	hypothetical protein POPTR_0003s16380g (*Populus trichocarpa*)
133	88.66176295	XP_002309105.2	hypothetical protein POPTR_0006s09540g (*Populus trichocarpa*)
134	88.40967508	XP_006373842.1	hypothetical protein POPTR_0016s07990g (*Populus trichocarpa*)
135	88.07662117	XP_006377135.1	hypothetical protein POPTR_0011s00430g (*Populus trichocarpa*)
136	88.04838915	XP_002302322.2	hypothetical protein POPTR_0002s10200g (*Populus trichocarpa*)
137	87.97837392	XP_002317632.1	hypothetical protein POPTR_0011s14830g (*Populus

			trichocarpa)
138	87.93969131	XP_006381812.1	AAA-type ATPase family protein (*Populus trichocarpa*)
139	87.564597	ADW95376.1	Kunitz-type trypsin inhibitor (*Populus nigra*)
140	87.41749534	XP_002299022.2	hypothetical protein POPTR_0001s46620g (*Populus trichocarpa*)
141	86.94001381	XP_006379156.1	pathogenesis-related family protein (*Populus trichocarpa*)
142	86.58016354	XP_011023016.1	PREDICTED: endogenous alpha-amylase/subtilisin inhibitor-like (*Populus euphratica*)
143	86.45766935	XP_011019763.1	PREDICTED: coatomer subunit beta'-2-like (*Populus euphratica*)
144	86.11189669	XP_002305291.2	hypothetical protein POPTR_0004s10610g (*Populus trichocarpa*)
145	85.46394336	XP_006368348.1	xyloglucan endotransglycosylase 2 family protein (*Populus trichocarpa*)
146	84.94578554	XP_002311689.2	proton-dependent oligopeptide transport family protein (*Populus trichocarpa*)
147	84.90872384	XP_002324034.1	hypothetical protein POPTR_0017s11350g (*Populus trichocarpa*)
148	84.72572023	XP_011025245.1	PREDICTED: LOW QUALITY PROTEIN: G-type lectin S-receptor-like serine/threonine-protein kinase RLK1 (*Populus euphratica*)
149	84.32970697	XP_002320914.1	hypothetical protein POPTR_0014s10440g (*Populus trichocarpa*)
150	83.62003367	XP_006384698.1	hypothetical protein POPTR_0004s20290g (*Populus trichocarpa*)
151	82.70309515	XP_002302215.1	calcium-binding family protein (*Populus trichocarpa*)
152	82.46604073	XP_006369422.1	hypothetical protein POPTR_0001s23070g (*Populus trichocarpa*)
153	82.39046705	XP_006377913.1	hypothetical protein POPTR_0011s16240g (*Populus trichocarpa*)
154	82.15085819	XP_002323542.2	hypothetical protein POPTR_0016s11600g (*Populus trichocarpa*)
155	81.98806796	XP_002307001.2	hypothetical protein POPTR_0005s27850g (*Populus trichocarpa*)
156	81.91685662	XP_002314193.1	hypothetical protein POPTR_0009s03360g (*Populus trichocarpa*)
157	81.90715455	XP_011009969.1	PREDICTED: protein LURP-one-related 15-like isoform X1 (*Populus euphratica*)
158	81.61436181	XP_006375881.1	hypothetical protein POPTR_0013s05110g (*Populus trichocarpa*)
159	81.53406183	XP_002306220.1	chitinase family protein (*Populus trichocarpa*)
160	81.40363708	XP_011043096.1	PREDICTED: E3 ubiquitin-protein ligase RING1-like (*Populus euphratica*)
161	81.33845607	XP_002304825.2	4-coumarate-CoA ligase family protein (*Populus*

			trichocarpa)
162	81.11641373	XP_002310378.1	hypothetical protein POPTR_0007s00370g (*Populus trichocarpa*)
163	80.49519346	XP_002299035.1	hypothetical protein POPTR_0001s46760g (*Populus trichocarpa*)
164	80.12273631	XP_002300292.2	Pathogenesis-related family protein (*Populus trichocarpa*)
165	80.04930571	XP_002299654.1	hypothetical protein POPTR_0001s23390g (*Populus trichocarpa*)
166	80.01684908	XP_006370473.1	hypothetical protein POPTR_0001s43040g (*Populus trichocarpa*)
167	79.78357299	XP_011010041.1	PREDICTED: SNAP25 homologous protein SNAP33-like isoform X1 (*Populus euphratica*)
168	78.98964634	XP_002316826.1	hypothetical protein POPTR_0011s07400g (*Populus trichocarpa*)
169	78.79441711	XP_002323569.2	hypothetical protein POPTR_0016s12140g (*Populus trichocarpa*)
170	78.75459561	XP_002299152.2	hypothetical protein POPTR_0001s05050g (*Populus trichocarpa*)
171	78.74694625	XP_002321484.2	hypothetical protein POPTR_0015s03860g (*Populus trichocarpa*)
172	78.62466412	XP_002318762.2	MATRIX METALLOproteinASE (*Populus trichocarpa*)
173	77.96543456	XP_002318542.2	calmodulin-binding protein 60-C (*Populus trichocarpa*)
174	77.95965539	XP_011013282.1	PREDICTED: E3 ubiquitin-protein ligase RGLG2-like isoform X1 (*Populus euphratica*)
175	77.50561654	XP_002302813.1	hypothetical protein POPTR_0002s22840g (*Populus trichocarpa*)
176	77.49325318	KRH56958.1	hypothetical protein GLYMA_05G029800 (*Glycine max*)
177	77.27519019	XP_006370773.1	FAD-binding domain-containing family protein (*Populus trichocarpa*)
178	77.22602731	XP_002299731.2	integral membrane family protein (*Populus trichocarpa*)
179	77.21039046	XP_011038566.1	PREDICTED: pathogenesis-related protein PR-4-like isoform X2 (*Populus euphratica*)
180	77.18885144	XP_006388365.1	hypothetical protein POPTR_0209s00220g (*Populus trichocarpa*)
181	77.18317797	XP_002320839.1	chitinase family protein (*Populus trichocarpa*)
182	76.53762831	XP_011007817.1	PREDICTED: beta-glucosidase 12-like (*Populus euphratica*)
183	76.22455793	XP_006383272.1	hypothetical protein POPTR_0005s13050g (*Populus trichocarpa*)
184	76.08992017	XP_006374862.1	hypothetical protein POPTR_0014s02160g (*Populus trichocarpa*)
185	75.90011348	XP_006385495.1	hypothetical protein POPTR_0003s05980g (*Populus trichocarpa*)
186	75.86701161	XP_006383697.1	hypothetical protein POPTR_0005s24230g (*Populus

			trichocarpa)
187	75.74364113	XP_002305405.1	allene oxide synthase family protein (*Populus trichocarpa*)
188	75.68131504	XP_002304154.2	hypothetical protein POPTR_0003s05970g (*Populus trichocarpa*)
189	75.66661851	XP_002322148.1	hypothetical protein POPTR_0015s08200g (*Populus trichocarpa*)

Table S10. Genes associated with plant defence in the JA-related module.

No.	IC value	Homologous protein ID	NR description
4	4.548884762	XP_002321141.2	LysM domain-containing receptor-like kinase 4 family protein (*Populus trichocarpa*)
39	2.242647187	XP_011017946.1	PREDICTED: uncharacterized protein LOC105121118 isoform X1 (*Populus euphratica*)
42	2.112950319	XP_002321444.2	hypothetical protein POPTR_0015s02070g (*Populus trichocarpa*)
45	1.990276881	XP_002304118.1	hypothetical protein POPTR_0003s06670g (*Populus trichocarpa*)
62	1.409799778	XP_002323692.2	hypothetical protein POPTR_0016s14870g (*Populus trichocarpa*)
71	1.295845583	XP_002303073.2	basic helix-loop-helix family protein (*Populus trichocarpa*)
98	0.857746219	XP_002313793.1	hypothetical protein POPTR_0009s11940g (*Populus trichocarpa*)
102	0.77820179	XP_006383530.1	WRKY transcription factor 15 family protein (*Populus trichocarpa*)
112	0.679365906	XP_002306849.2	hypothetical protein POPTR_0005s24550g (*Populus trichocarpa*)
123	0.545177325	XP_006373671.1	hypothetical protein POPTR_0016s02670g (*Populus trichocarpa*)
128	0.501605946	XP_002314737.1	phytosulfokine receptor precursor family protein (*Populus trichocarpa*)
152	0.26638782	XP_002320933.2	Endochitinase 2 family protein (*Populus trichocarpa*)

Table S11. Genes associated with plant defence in the bound SA-related module.

No.	IC value	Homologous protein ID	NR Description
26	0.783569409	XP_006375889.1	hypothetical protein POPTR_0013s05310g (*Populus trichocarpa*)
51	0.162044544	XP_006385777.1	NADPH oxidase family protein (*Populus trichocarpa*)
52	0.155412956	XP_002312455.2	calcium binding family protein (*Populus trichocarpa*)
57	0.117215567	XP_011042788.1	PREDICTED: transcription factor bHLH128-like (*Populus euphratica*)

Table S12. Genes associated with plant defence in the free SA-related module.

No.	IC value	Homologous protein ID	NR description
28	120.8475033	XP_006383679.1	hypothetical protein POPTR_0005s23720g (*Populus trichocarpa*)
32	118.2384071	XP_002324257.1	K+ rectifying channel family protein (*Populus trichocarpa*)
54	110.595504	XP_002323865.1	hypothetical protein POPTR_0017s12100g (*Populus trichocarpa*)
57	110.1107081	XP_002316607.1	hypothetical protein POPTR_0011s00410g (*Populus trichocarpa*)
93	99.1749001	XP_006387602.1	hypothetical protein POPTR_0790s00210g (*Populus trichocarpa*)
118	91.99540428	XP_006373216.1	hypothetical protein POPTR_0017s09750g (*Populus trichocarpa*)
135	88.07662117	XP_006377135.1	hypothetical protein POPTR_0011s00430g (*Populus trichocarpa*)
141	86.94001381	XP_006379156.1	pathogenesis-related family protein (*Populus trichocarpa*)
151	82.70309515	XP_002302215.1	calcium-binding family protein (*Populus trichocarpa*)
159	81.53406183	XP_002306220.1	chitinase family protein (*Populus trichocarpa*)
164	80.12273631	XP_002300292.2	Pathogenesis-related family protein (*Populus trichocarpa*)
175	77.50561654	XP_002302813.1	hypothetical protein POPTR_0002s22840g (*Populus trichocarpa*)
179	77.21039046	XP_011038566.1	PREDICTED: pathogenesis-related protein PR-4-like isoform X2 (*Populus euphratica*)
195	74.7420434	XP_006387602.1	hypothetical protein POPTR_0790s00210g (*Populus trichocarpa*)
196	74.55316647	XP_002306659.1	calcium-binding family protein (*Populus trichocarpa*)
200	73.66890421	XP_011036106.1	PREDICTED: probable WRKY transcription factor 75 (*Populus euphratica*)
204	72.80582519	XP_002321477.2	hypothetical protein POPTR_0015s03770g (*Populus trichocarpa*)
220	68.51934477	XP_002303770.1	hypothetical protein POPTR_0003s16580g (*Populus trichocarpa*)
228	66.70627763	ABK94784.1	unknown (*Populus trichocarpa*)
232	65.85789096	XP_002305730.1	hypothetical protein POPTR_0004s05920g (*Populus trichocarpa*)
237	65.26983883	ADP69172.1	pathogenesis related protein-1 (*Populus tomentosa*)
242	64.08435545	XP_002299864.1	hypothetical protein POPTR_0001s24880g (*Populus trichocarpa*)
251	63.17369484	XP_006369712.1	hypothetical protein POPTR_0001s29540g (*Populus trichocarpa*)
257	61.96880658	XP_002325280.1	hypothetical protein POPTR_0019s00980g (*Populus trichocarpa*)
286	56.42464725	XP_006381051.1	polygalacturonase inhibiting family protein (*Populus*

			trichocarpa)
297	53.54928705	ABF81442.1	NBS-LRR type disease resistance protein (*Populus trichocarpa*)
300	52.92138307	XP_006389390.1	hypothetical protein POPTR_0025s00270g (*Populus trichocarpa*)
304	52.21008024	XP_002323262.1	Calmodulin-like family protein (*Populus trichocarpa*)
305	51.63109975	XP_011047157.1	PREDICTED: probable WRKY transcription factor 72 (*Populus euphratica*)
341	46.26933536	XP_002308958.1	calcium-dependent protein kinase (*Populus trichocarpa*)
342	46.22514919	XP_002322442.1	putative esterase family protein (*Populus trichocarpa*)
371	43.12610329	XP_006372565.1	myb family transcription factor family protein (*Populus trichocarpa*)
380	42.31868736	XP_011015511.1	PREDICTED: probable leucine-rich repeat receptor-like protein kinase At1g35710 isoform X1 (*Populus euphratica*)
386	41.64267491	XP_011009761.1	PREDICTED: LRR receptor-like serine/threonine-protein kinase FLS2 isoform X1 (*Populus euphratica*)
394	40.70620812	XP_006385291.1	glycerol kinase family protein (*Populus trichocarpa*)
395	40.639853	XP_006389342.1	hypothetical protein POPTR_0027s00200g (*Populus trichocarpa*)
411	39.46024161	XP_006385240.1	hypothetical protein POPTR_0003s02030g (*Populus trichocarpa*)
428	37.82865827	XP_011018453.1	PREDICTED: disease resistance protein RPM1-like (*Populus euphratica*)
438	37.2495817	XP_002304741.2	hypothetical protein POPTR_0003s16750g (*Populus trichocarpa*)
459	35.6187444	XP_002313167.2	hypothetical protein POPTR_0009s09390g (*Populus trichocarpa*)
461	35.5276817	XP_011033661.1	PREDICTED: probable indole-3-acetic acid-amido synthetase GH3.6 (*Populus euphratica*)
466	35.06563212	XP_002309231.2	hypothetical protein POPTR_0006s15760g (*Populus trichocarpa*)
476	34.12659493	XP_002297728.1	hsr203J family protein (*Populus trichocarpa*)
479	33.9678179	XP_011007196.1	PREDICTED: chitin elicitor receptor kinase 1-like isoform X1 (*Populus euphratica*)
499	31.94411704	XP_006382336.1	hypothetical protein POPTR_0005s01150g (*Populus trichocarpa*)
506	31.2957153	XP_011018453.1	PREDICTED: disease resistance protein RPM1-like (*Populus euphratica*)
509	31.14320615	XP_002302808.1	WRKY transcription factor 47 family protein (*Populus trichocarpa*)
516	30.49046349	ABF81442.1	NBS-LRR type disease resistance protein (*Populus trichocarpa*)
536	28.84511111	XP_006380674.1	hypothetical protein POPTR_0007s10350g (*Populus*

			trichocarpa)
543	28.18601891	XP_002322142.1	enhanced disease susceptibility 1 family protein (*Populus trichocarpa*)
558	26.84096687	XP_002300193.2	hypothetical protein POPTR_0001s31820g, partial (*Populus trichocarpa*)
579	25.82166172	XP_006369852.1	hypothetical protein POPTR_0001s34030g (*Populus trichocarpa*)
582	25.76591877	XP_006374251.1	mitogen-activated protein kinase kinase (*Populus trichocarpa*)
592	25.16482998	XP_002315937.2	hypothetical protein POPTR_0010s13360g (*Populus trichocarpa*)
595	25.06200247	XP_006382321.1	hypothetical protein POPTR_0005s01010g, partial (*Populus trichocarpa*)
599	25.0155853	XP_002311750.2	hypothetical protein POPTR_0008s184201g, partial (*Populus trichocarpa*)
615	24.23732173	XP_002312923.2	Chain A family protein (*Populus trichocarpa*)
626	23.47694776	XP_002313936.2	pathogenesis-related family protein (*Populus trichocarpa*)
641	22.49225624	XP_002312322.1	hypersensitive reaction associated Ca2+-binding family protein (*Populus trichocarpa*)
653	21.6127707	XP_002302045.1	GmMYB29 family protein (*Populus trichocarpa*)
665	20.92802851	XP_006382382.1	hypothetical protein POPTR_0005s01610g (*Populus trichocarpa*)
690	19.53603974	XP_002306976.2	catalase family protein (*Populus trichocarpa*)
692	19.49318899	XP_002310610.2	hypothetical protein POPTR_0007s06720g (*Populus trichocarpa*)
699	19.32932604	XP_006382787.1	calmodulin-like protein 6a (*Populus trichocarpa*)
722	18.5920426	XP_002323299.2	hypothetical protein POPTR_0016s05100g (*Populus trichocarpa*)
745	17.18310314	XP_002310978.1	calcium-dependent protein kinase 2 (*Populus trichocarpa*)
755	16.73798153	XP_002301429.2	auxin-responsive family protein (*Populus trichocarpa*)
797	15.26149656	XP_002307775.2	hypothetical protein POPTR_0005s27070g (*Populus trichocarpa*)
842	13.51184245	XP_002319076.1	HEV1.2 family protein (*Populus trichocarpa*)
861	13.09997196	XP_006368770.1	hypothetical protein POPTR_0001s09970g (*Populus trichocarpa*)
867	12.97836344	XP_002302075.1	auxin-induced protein aux28 (*Populus trichocarpa*)
870	12.90942345	XP_002323001.2	hypothetical protein POPTR_0016s12810g (*Populus trichocarpa*)
890	12.21280118	XP_006368520.1	hypothetical protein POPTR_0001s03710g (*Populus trichocarpa*)
904	11.71205976	XP_002324703.2	hypothetical protein POPTR_0018s14150g (*Populus trichocarpa*)
931	10.67799805	XP_002323024.1	hypothetical protein POPTR_0016s13320g (*Populus trichocarpa*)

933	10.62517782	XP_002305278.1	putative protein phosphatase (*Populus trichocarpa*)
952	9.920251584	XP_006389367.1	hypothetical protein POPTR_0027s00410g (*Populus trichocarpa*)
955	9.811474487	XP_002305198.1	hypothetical protein POPTR_0004s08890g (*Populus trichocarpa*)
1002	8.474837832	XP_006374229.1	calmodulin-like protein 6a (*Populus trichocarpa*)
1004	8.430551795	XP_002303610.1	hypothetical protein POPTR_0003s13250g (*Populus trichocarpa*)
1008	8.339292507	XP_002320254.2	hypothetical protein POPTR_0014s10750g (*Populus trichocarpa*)
1036	7.554245143	XP_002302351.1	auxin-responsive family protein (*Populus trichocarpa*)
1051	7.287905102	XP_002305730.1	hypothetical protein POPTR_0004s05920g (*Populus trichocarpa*)
1070	6.861443162	XP_002317397.1	WRKY transcription factor 65 family protein (*Populus trichocarpa*)
1076	6.780567708	XP_002301269.1	hypothetical protein POPTR_0002s14550g (*Populus trichocarpa*)
1082	6.654214232	XP_006375351.1	hypothetical protein POPTR_0014s08600g (*Populus trichocarpa*)
1114	6.138278808	XP_002314327.2	hypothetical protein POPTR_0010s00390g (*Populus trichocarpa*)
1133	5.920144683	XP_002298742.1	C2 domain-containing family protein (*Populus trichocarpa*)
1164	5.414907325	XP_002320126.1	kinase family protein (*Populus trichocarpa*)
1172	5.307917742	XP_002314171.2	hypothetical protein POPTR_0009s03800g (*Populus trichocarpa*)
1177	5.264554607	XP_002306615.1	amino acid permease family protein (*Populus trichocarpa*)
1180	5.250122045	XP_002300854.2	hypothetical protein POPTR_0002s05590g (*Populus trichocarpa*)
1202	4.964140576	XP_002300570.2	hypothetical protein POPTR_0001s47050g (*Populus trichocarpa*)
1208	4.931313422	XP_011005453.1	PREDICTED: receptor-like protein 12 isoform X3 (*Populus euphratica*)
1226	4.686310699	XP_002322129.1	calcium-dependent protein kinase (*Populus trichocarpa*)
1270	4.065943252	XP_006389344.1	hypothetical protein POPTR_0027s00220g (*Populus trichocarpa*)
1290	3.848124983	XP_006384452.1	hypothetical protein POPTR_0004s15220g (*Populus trichocarpa*)
1314	3.549353238	XP_002318373.1	hypothetical protein POPTR_0012s01380g (*Populus trichocarpa*)
1391	2.871763128	XP_002305263.1	hypothetical protein POPTR_0004s07190g (*Populus trichocarpa*)
1396	2.822597637	XP_002306180.1	myb family transcription factor family protein (*Populus trichocarpa*)

1415	2.702537572	XP_002312507.1	phytosulfokine receptor precursor family protein (*Populus trichocarpa*)
1465	2.265510656	XP_002308264.1	hypothetical protein POPTR_0006s11150g (*Populus trichocarpa*)
1472	2.203829075	CAH56500.1	putative histidine-containing phosphotransfer protein 2 (*Populus* × *canadensis*)
1476	2.161108756	XP_002304048.2	bZIP family transcription factor family protein (*Populus trichocarpa*)
1477	2.160091082	XP_002317717.1	calmodulin-like protein 6a (*Populus trichocarpa*)
1481	2.127216664	XP_006385188.1	auxin response factor 1 family protein (*Populus trichocarpa*)
1494	2.027303877	XP_002315736.1	aux/IAA family protein (*Populus trichocarpa*)
1497	2.002219366	XP_002301514.2	hypothetical protein POPTR_0002s19630g (*Populus trichocarpa*)
1503	1.956756672	XP_011023935.1	PREDICTED: protein TIFY 6B-like isoform X1 (*Populus euphratica*)
1505	1.937251373	BAA94600.1	1-aminocyclopropane-1-carboxylate synthase (*Populus* × *canadensis*)
1514	1.810522447	XP_002298064.1	hypothetical protein POPTR_0001s08770g (*Populus trichocarpa*)
1518	1.784771931	XP_002320183.1	hypothetical protein POPTR_0014s09120g (*Populus trichocarpa*)
1529	1.699809892	XP_011005376.1	PREDICTED: receptor-like protein 12 (*Populus euphratica*)
1533	1.689317551	XP_002301811.1	kinase family protein (*Populus trichocarpa*)
1543	1.648861031	XP_002313138.1	hypothetical protein POPTR_0009s10070g (*Populus trichocarpa*)
1557	1.597292748	XP_002306828.2	basic helix-loop-helix family protein (*Populus trichocarpa*)
1583	1.490555166	XP_002300248.2	auxin-responsive GH3 family protein (*Populus trichocarpa*)
1588	1.469734894	XP_002304822.2	hypothetical protein POPTR_0003s18900g (*Populus trichocarpa*)
1603	1.386358737	XP_006374887.1	hypothetical protein POPTR_0014s02420g (*Populus trichocarpa*)
1622	1.26352022	XP_006382369.1	hypothetical protein POPTR_0005s01500g, partial (*Populus trichocarpa*)
1644	1.195659659	XP_002302066.1	basic helix-loop-helix family protein (*Populus trichocarpa*)
1709	0.84339733	XP_002307983.2	Two-component response regulator ARR8 family protein (*Populus trichocarpa*)
1712	0.823806293	XP_011038575.1	PREDICTED: transcription factor UNE12-like (*Populus euphratica*)
1731	0.748924425	XP_002310503.1	hypothetical protein POPTR_0007s03770g (*Populus trichocarpa*)
1750	0.686422554	XP_006385006.1	hypothetical protein POPTR_0004s23000g (*Populus*

			trichocarpa)
1753	0.674363881	XP_006381001.1	calcium-binding EF hand family protein (*Populus trichocarpa*)
1756	0.657409933	XP_002310398.2	mitogen-activated protein kinase 7 (*Populus trichocarpa*)
1799	0.446581679	XP_002302637.2	basic helix-loop-helix family protein (*Populus trichocarpa*)
1819	0.384987523	XP_002324788.2	hypothetical protein POPTR_0018s08270g (*Populus trichocarpa*)
1832	0.34010324	XP_002301020.2	hypothetical protein POPTR_0002s09050g (*Populus trichocarpa*)
1876	0.174475073	XP_006385006.1	hypothetical protein POPTR_0004s23000g (*Populus trichocarpa*)
1889	0.101565819	XP_011028179.1	PREDICTED: transcription factor MYC2-like (*Populus euphratica*)

Table S13. KEGG enrichment for hub genes in JA-related module.

KEGG A class	KEGG B class	Pathway	Amount	Pathway ID	Gene No.	K IDs
Metabolism	Global and overview maps	Metabolic pathways	4	ko01100	3, 14, 9, 1	K14652, K01583, K01772, K16055
Metabolism	Global and overview maps	Biosynthesis of secondary metabolites	4	ko01110	3, 11, 9, 10	K14652, K08241, K01772, K12153
Metabolism	Amino acid metabolism	Arginine and proline metabolism	1	ko00330	14	K01583
Metabolism	Metabolism of other amino acids	Cyanoamino acid metabolism	1	ko00460	10	K12153
Metabolism	Carbohydrate metabolism	Starch and sucrose metabolism	1	ko00500	1	K16055
Metabolism	Lipid metabolism	alpha-Linolenic acid metabolism	1	ko00592	11	K08241
Metabolism	Metabolism of cofactors and vitamins	Riboflavin metabolism	1	ko00740	3	K14652
Metabolism	Metabolism of cofactors and vitamins	Folate biosynthesis	1	ko00790	3	K14652
Metabolism	Metabolism of cofactors and vitamins	Porphyrin and chlorophyll metabolism	1	ko00860	9	K01772
Metabolism	Biosynthesis of other secondary metabolites	Glucosinolate biosynthesis	1	ko00966	10	K12153
Metabolism	Global and overview maps	2-Oxocarboxylic acid metabolism	1	ko01210	10	K12153
Genetic Information Processing	Replication and repair	Mismatch repair	1	ko03430	13	K07456
Organismal Systems	Environmenta l adaptation	Plant-pathogen interaction	1	ko04626	4	K13429

Table S14. KEGG enrichment for hub genes in bound SA-related module.

KEGG A class	KEGG B class	Pathway	Amount	Pathway ID	Gene No.	K IDs
Metabolism	Lipid metabolism	Ether lipid metabolism	1	ko00565	4	K04628
Metabolism	Lipid metabolism	Sphingolipid metabolism	1	ko00600	4	K04628
Metabolism	Global and overview maps	Metabolic pathways	1	ko01100	4	K04628

Table S15. KEGG enrichment for hub genes in free SA-related module.

KEGG A class	KEGG B class	Pathway	Amount	Pathway ID	Gene No.	K IDs
Metabolism	Global and overview maps	Metabolic pathways	29	ko01100	93, 159, 179, 59, 80, 41, 108, 127, 169, 161, 187, 53, 97, 152, 182, 13, 181, 91, 31, 7, 55, 78, 17, 114, 124, 170, 2, 144, 30	K20547, K20547, K20547, K15920, K13066, K07437, K07437, K07418, K01988, K01904, K01723, K01188, K01188, K01188, K01188, K01183, K01183, K01176, K01054, K01051, K01051, K01051, K00511, K00430, K00430, K00430, K00423, K00318, K00021
Metabolism	Global and overview maps	Biosynthesis of secondary metabolites	17	ko01110	123, 122, 80, 84, 89, 161, 187, 53, 97, 152, 182, 17, 114, 124, 170, 144, 30	K15813, K13081, K13066, K08233, K03809, K01904, K01723, K01188, K01188, K01188, K01188, K00511, K00430, K00430, K00430, K00318, K00021
Metabolism	Biosynthesis of other secondary metabolites	Phenylpropanoid biosynthesis	9	ko00940	80, 161, 53, 97, 152, 182, 114, 124, 170	K13066, K01904, K01188, K01188, K01188, K01188, K00430, K00430, K00430
Environmental Information Processing	Signal transduction	MAPK signaling pathway - plant	8	ko04016	93, 159, 179, 57, 135, 141, 164, 54	K20547, K20547, K20547, K14496, K14496, K13449, K13449, K02183
Metabolism	Carbohydrate metabolism	Starch and sucrose metabolism	7	ko00500	52, 61, 53, 97, 152, 182, 91	K19892, K19892, K01188, K01188, K01188, K01188, K01176
Organismal Systems	Environmental adaptation	Plant-pathogen interaction	7	ko04626	118, 141, 164, 28, 151, 32,	K18835, K13449, K13449, K13448, K13448, K05391,

					54	K02183
Metabolism	Carbohydrate metabolism	Amino sugar and nucleotide sugar metabolism	6	ko00520	93, 159, 179, 59, 13, 181	K20547, K20547, K20547, K15920, K01183, K01183
Environmental Information Processing	Signal transduction	Plant hormone signal transduction	5	ko04075	57, 135, 175, 141, 164	K14496, K14496, K14493, K13449, K13449
Metabolism	Metabolism of other amino acids	Cyanoamino acid metabolism	4	ko00460	53, 97, 152, 182	K01188, K01188, K01188, K01188
Metabolism	Carbohydrate metabolism	Pentose and glucuronate interconversions	3	ko00040	7, 55, 78	K01051, K01051, K01051
Metabolism	Metabolism of terpenoids and polyketides	Sesquiterpenoid and triterpenoid biosynthesis	3	ko00909	123, 42, 17	K15813, K15803, K00511
Genetic Information Processing	Folding, sorting and degradation	Protein processing in endoplasmic reticulum	3	ko04141	87, 103, 65	K14006, K14006, K03283
Metabolism	Metabolism of cofactors and vitamins	Ubiquinone and other terpenoid-quinone biosynthesis	2	ko00130	89, 161	K03809, K01904
Genetic Information Processing	Folding, sorting and degradation	RNA degradation	2	ko03018	158, 11	K12581, K12581
Genetic Information Processing	Folding, sorting and degradation	Ubiquitin mediated proteolysis	2	ko04120	19, 112	K10260, K10260
Cellular Processes	Transport and catabolism	Endocytosis	2	ko04144	106, 65	K05747, K03283
Metabolism	Carbohydrate metabolism	Ascorbate and aldarate metabolism	1	ko00053	2	K00423
Metabolism	Lipid metabolism	Steroid biosynthesis	1	ko00100	17	K00511
Metabolism	Amino acid metabolism	Arginine and proline metabolism	1	ko00330	144	K00318
Metabolism	Amino acid metabolism	Phenylalanine metabolism	1	ko00360	161	K01904
Metabolism	Glycan biosynthesis and metabolism	Other glycan degradation	1	ko00511	113	K01206
Metabolism	Lipid metabolism	Glycerolipid metabolism	1	ko00561	31	K01054
Metabolism	Lipid metabolism	Arachidonic acid metabolism	1	ko00590	127	K07418
Metabolism	Lipid metabolism	Linoleic acid metabolism	1	ko00591	127	K07418

Metabolism	Lipid metabolism	alpha-Linolenic acid metabolism	1	ko00592	187	K01723
Metabolism	Glycan biosynthesis and metabolism	Glycosphingolipid biosynthesis - lacto and neolacto series	1	ko00601	169	K01988
Metabolism	Glycan biosynthesis and metabolism	Glycosphingolipid biosynthesis - globo and isoglobo series	1	ko00603	169	K01988
Metabolism	Carbohydrate metabolism	Pyruvate metabolism	1	ko00620	76	K18881
Metabolism	Metabolism of terpenoids and polyketides	Terpenoid backbone biosynthesis	1	ko00900	30	K00021
Metabolism	Biosynthesis of other secondary metabolites	Flavonoid biosynthesis	1	ko00941	122	K13081
Genetic Information Processing	Translation	Aminoacyl-tRNA biosynthesis	1	ko00970	58	K01870
Environmental Information Processing	Membrane transport	ABC transporters	1	ko02010	71	K05658
Genetic Information Processing	Replication and repair	DNA replication	1	ko03030	9	K07466
Genetic Information Processing	Transcription	Spliceosome	1	ko03040	65	K03283
Genetic Information Processing	Folding, sorting and degradation	Proteasome	1	ko03050	100	K06700
Genetic Information Processing	Replication and repair	Nucleotide excision repair	1	ko03420	9	K07466
Genetic Information Processing	Replication and repair	Mismatch repair	1	ko03430	9	K07466
Genetic Information Processing	Replication and repair	Homologous recombination	1	ko03440	9	K07466
Environmental Information Processing	Signal transduction	Phosphatidylinositol signaling system	1	ko04070	54	K02183
Organismal	Environmental	Circadian rhythm -	1	ko04712	168	K16222

Systems	adaptation	plant

Table S16. Expression characteristics of plant defence-related genes in the JA-related module.

IC No.	GS	GS *p*-value	MM	MM *p*-value	Homologous protein ID	NR Description	Expression characteristics in compatible breed 'Intolerant'
2	0.76	0.00	0.90	0.00	XP_002307385.1	VQ motif-containing family protein	up-regulated at 2 hpi
10	0.80	0.00	0.82	0.00	AHF20912.1	cytochrome P450	up-regulated at 2 hpi
11	0.74	0.00	0.83	0.00	XP_002307671.1	jasmonic acid carboxyl methyltransferase family protein	up-regulated at 2 hpi
22	0.58	0.00	0.83	0.00	XP_011006301.1	PREDICTED: target of Myb protein 1-like	up-regulated at 2 hpi
34	0.63	0.00	0.84	0.00	XP_002305655.1	F-box family protein	up-regulated at 2 hpi
37	0.59	0.00	0.82	0.00	XP_006369133.1	lipoxygenase family protein	up-regulated at 2 hpi
51	0.42	0.01	0.73	0.00	XP_002301587.1	auxin-responsive family protein	up-regulated at 2 hpi
71	0.48	0.00	0.76	0.00	XP_002303073.2	basic helix-loop-helix family protein	up-regulated at 2 hpi,
72	0.56	0.00	0.74	0.00	XP_002302856.1	1-aminocyclopropane-1-carboxylate oxidase family protein	up-regulated at 2 hpi
75	0.64	0.00	0.81	0.00	XP_002314993.1	S-adenosylmethionine synthase family protein	up-regulated at 2 hpi
102	0.53	0.00	0.75	0.00	XP_006383530.1	WRKY transcription factor 15 family protein	up-regulated at 2 hpi
4	0.69	0.00	0.92	0.00	XP_002321141.2	LysM domain-containing receptor-like kinase 4 family protein	down-regulated at 2 dpi and 4 dpi
6	0.57	0.00	0.88	0.00	XP_002324557.1	calmodulin-binding protein 60-D	down-regulated at 2 dpi and 4 dpi
22	0.58	0.00	0.83	0.00	XP_011006301.1	PREDICTED: target of Myb protein 1-like	down-regulated at 2 dpi and 4 dpi
35	0.74	0.00	0.84	0.00	XP_002306205.2	putative glucosyltransferase family protein	unchanged at 1 dpi and 2dpi
51	0.42	0.01	0.73	0.00	XP_002301587.1	auxin-responsive family protein	down-regulated at 2 dpi and 4dpi
70	0.55	0.00	0.76	0.00	XP_006379232.1	Cf-4/9 disease resistance-like family protein	down-regulated at 2 dpi and 4dpi
72	0.56	0.00	0.74	0.00	XP_002302856.1	1-aminocyclopropane-1-carboxylate oxidase family protein	down-regulated at 2 dpi
84	0.35	0.02	0.62	0.00	XP_011027208.1	PREDICTED: elicitor-responsive protein 1-like	down-regulated at 2 dpi and 4dpi
128	0.53	0.00	0.71	0.00	XP_002314737.1	phytosulfokine receptor precursor family protein	down-regulated at 2 dpi and 4dpi
2	0.76	0.00	0.90	0.00	XP_002307385.1	VQ motif-containing family protein	up-regulated at 4 dpi
34	0.63	0.00	0.84	0.00	XP_002305655.1	F-box family protein	up-regulated at 4 dpi
102	0.53	0.00	0.75	0.00	XP_006383530.1	WRKY transcription factor 15 family protein	up-regulated at 12 hpi and 1dpi
152	0.37	0.02	0.63	0.00	XP_002320933.2	Endochitinase 2 family protein	up-regulated at 4 dpi

Table S17. Expression characteristics of plant defence-related genes in the bound SA-related module.

IC No.	GS_{all}	GS_{all} *p*-value	GS_{bound}	GS_{bound} *p*-value	MM	MM *p*-value	Homologous protein ID	NR Description	Expression characteristics in compatible breed 'Intolerant'
1	0.42	0.01	0.42	0.01	0.96	0.00	XP_002307385.1	NBS-LRR resistance gene-like protein ARGH30	down-regulated at 4 dpi
5	0.42	0.01	0.42	0.01	0.94	0.00	AHF20912.1	NBS-LRR resistance gene-like protein ARGH30	down-regulated at 4 dpi
37	0.29	0.06	0.29	0.06	0.67	0.00	XP_002307671.1	cytochrome P450 family protein	up-regulated at 4 dpi

Table S18. Expression characteristics of plant defence-related genes in the free SA-related module.

IC No.	GS	GS *p*-value	MM	MM *p*-value	Homologous protein ID	NR Description	Expression characteristics in compatible breed 'Intolerant'
16	0.40	0.01	0.88	0.00	XP_002306491.1	cytochrome P450 family protein	down-regulated at 4 dpi
32	0.48	0.00	0.91	0.00	XP_002324257.1	K+ rectifying channel family protein	down-regulated at 4 dpi
41	0.43	0.00	0.92	0.00	XP_011007351.1	PREDICTED: cytochrome P450 87A3-like	down-regulated at 4 dpi
79	0.43	0.00	0.88	0.00	ADW95387.1	Kunitz-type trypsin inhibitor	down-regulated at 4 dpi
139	0.27	0.09	0.83	0.00	ADW95376.1	Kunitz-type trypsin inhibitor	down-regulated at 4 dpi
141	0.32	0.04	0.87	0.00	XP_006379156.1	pathogenesis-related family protein	down-regulated at 4 dpi
164	0.24	0.13	0.82	0.00	XP_002300292.2	Pathogenesis-related family protein	down-regulated at 4 dpi
187	0.41	0.01	0.91	0.00	XP_002305405.1	allene oxide synthase family protein	down-regulated at 4 dpi
192	0.31	0.05	0.85	0.00	XP_006375029.1	putative cytochrome P450 family protein	down-regulated at 4 dpi
195	0.29	0.06	0.86	0.00	ABK96628.1	unknown	down-regulated at 4 dpi
207	0.43	0.00	0.89	0.00	XP_002310288.1	cytochrome P450 family protein	down-regulated at 4 dpi
224	0.36	0.02	0.87	0.00	ACZ67171.1	plant disease resistance response protein, partial	down-regulated at 4 dpi
228	0.32	0.04	0.90	0.00	ABK94784.1	unknown	down-regulated at 4 dpi
234	0.30	0.05	0.84	0.00	XP_002309865.2	cytochrome P450 family protein	down-regulated at 4 dpi
274	0.44	0.00	0.83	0.00	XP_002320540.2	subtilisin-like protease family protein	down-regulated at 4 dpi
275	0.35	0.02	0.87	0.00	XP_011007635.1	PREDICTED: cysteine-rich receptor-like protein kinase 10	down-regulated at 4 dpi
305	0.42	0.01	0.80	0.00	XP_011047157.1	PREDICTED: probable WRKY transcription factor 72	down-regulated at 4 dpi
333	0.30	0.05	0.82	0.00	XP_002306490.1	cytochrome P450 family protein	down-regulated at 4 dpi
335	0.42	0.01	0.88	0.00	XP_002306682.1	putative pathogenesis-related family protein	down-regulated at 4 dpi
364	0.26	0.09	0.78	0.00	XP_011007635.1	PREDICTED: cysteine-rich receptor-like protein kinase 10	down-regulated at 4 dpi
371	0.27	0.09	0.82	0.00	XP_006372565.1	myb family transcription factor family protein	down-regulated at 4 dpi
376	0.34	0.03	0.70	0.00	ADW95389.1	Kunitz-type trypsin inhibitor	down-regulated at 4 dpi
386	0.30	0.05	0.82	0.00	XP_011009761.1	PREDICTED: LRR receptor-like serine/threonine-protein kinase FLS2 isoform X1	down-regulated at 4 dpi
390	0.32	0.04	0.87	0.00	XP_002309671.1	Stromal cell-derived factor 2-like protein precursor	down-regulated at 4 dpi
391	0.44	0.00	0.87	0.00	XP_006376441.1	C2 domain-containing family protein	down-regulated at 4 dpi
394	0.42	0.01	0.86	0.00	XP_006385291.1	glycerol kinase family protein	down-regulated at 4 dpi
403	0.46	0.00	0.90	0.00	XP_006368952.1	Bax inhibitor-1 family protein	down-regulated at 4 dpi
441	0.14	0.38	0.77	0.00	XP_002297684.2	wall-associated kinase family protein	down-regulated at 4 dpi
461	0.34	0.03	0.88	0.00	XP_011033661.1	PREDICTED: probable indole-3-acetic acid-amido synthetase GH3.6	down-regulated at 4 dpi
509	0.34	0.03	0.83	0.00	XP_002302808.1	WRKY transcription factor 47 family protein	down-regulated at 4 dpi
531	0.11	0.49	0.70	0.00	XP_002297684.2	wall-associated kinase family protein	down-regulated at 4 dpi
585	0.31	0.05	0.73	0.00	XP_002324752.1	leucine-rich repeat family protein	down-regulated at 4 dpi
591	0.34	0.03	0.74	0.00	XP_006384770.1	F-box family protein	down-regulated at 4 dpi
615	0.35	0.03	0.83	0.00	XP_002312923.2	Chain A family protein	down-regulated at 4 dpi
626	0.15	0.35	0.78	0.00	XP_002313936.2	pathogenesis-related family protein	down-regulated at 4 dpi
636	0.33	0.03	0.82	0.00	XP_002324926.2	VQ motif-containing family protein	down-regulated at 4 dpi
647	0.14	0.37	0.73	0.00	XP_011031755.1	PREDICTED: patatin-like protein 6 isoform X2	down-regulated at 4 dpi
723	0.31	0.04	0.76	0.00	XP_002298729.1	caffeoyl-CoA O-methyltransferase family protein	down-regulated at 4 dpi

760	0.42	0.01	0.65	0.00	XP_002309324.1	class V chitinase family protein	down-regulated at 4 dpi
842	0.34	0.03	0.76	0.00	XP_002319076.1	HEV1.2 family protein	down-regulated at 4 dpi
848	0.23	0.14	0.77	0.00	XP_006383016.1	F-box family protein	down-regulated at 4 dpi
933	0.33	0.03	0.75	0.00	XP_002305278.1	putative protein phosphatase	down-regulated at 4 dpi
1070	0.27	0.08	0.66	0.00	XP_002317397.1	WRKY transcription factor 65 family protein	down-regulated at 4 dpi
1133	0.25	0.12	0.64	0.00	XP_002298742.1	C2 domain-containing family protein	down-regulated at 4 dpi
1171	0.35	0.02	0.63	0.00	XP_002310007.2	cytochrome P450 family protein	down-regulated at 4 dpi
1177	-0.26	0.10	-0.60	0.00	XP_002306615.1	amino acid permease family protein	down-regulated at 4 dpi
1195	0.15	0.34	0.74	0.00	XP_002317572.1	non race-specific disease resistance 1 family protein	down-regulated at 4 dpi
1201	0.18	0.24	0.60	0.00	XP_011021732.1	PREDICTED: allene oxide cyclase 3, chloroplastic-like	down-regulated at 4 dpi
1208	0.50	0.00	0.67	0.00	XP_011005453.1	PREDICTED: receptor-like protein 12 isoform X3	down-regulated at 4 dpi
1407	0.21	0.19	0.51	0.00	XP_002308233.2	lectin protein kinase	down-regulated at 4 dpi
1415	0.29	0.06	0.50	0.00	XP_002312507.1	phytosulfokine receptor precursor family protein	down-regulated at 4 dpi
1529	0.06	0.70	0.53	0.00	XP_011005376.1	PREDICTED: receptor-like protein 12	down-regulated at 4 dpi
1684	0.25	0.11	0.49	0.00	XP_002310677.1	leucine-rich repeat family protein	down-regulated at 4 dpi
1709	-0.08	0.60	0.40	0.01	XP_002307983.2	Two-component response regulator ARR8 family protein	down-regulated at 4 dpi
1712	0.20	0.21	0.48	0.00	XP_011038575.1	PREDICTED: transcription factor UNE12-like	down-regulated at 4 dpi
1724	0.33	0.04	0.49	0.00	XP_011029415.1	PREDICTED: phytohormone-binding protein-like	down-regulated at 4 dpi
1753	0.23	0.13	0.40	0.01	XP_006381001.1	calcium-binding EF hand family protein	down-regulated at 4 dpi
1879	0.16	0.31	0.33	0.04	XP_002314220.1	F-box family protein	down-regulated at 4 dpi
196	0.27	0.09	0.87	0.00	XP_002306659.1	calcium-binding family protein	up-regulated at 2 hpi
286	0.40	0.01	0.80	0.00	XP_006381051.1	polygalacturonase inhibiting family protein	up-regulated at 2 hpi
333	0.30	0.05	0.82	0.00	XP_002306490.1	cytochrome P450 family protein	up-regulated at 2 hpi
461	0.34	0.03	0.88	0.00	XP_011033661.1	PREDICTED: probable indole-3-acetic acid-amido synthetase GH3.6	up-regulated at 2 hpi
476	0.26	0.10	0.75	0.00	XP_002297728.1	hsr203J family protein	up-regulated at 2 hpi
531	0.11	0.49	0.70	0.00	XP_002297684.2	wall-associated kinase family protein	up-regulated at 2 hpi
653	0.15	0.35	0.76	0.00	XP_002302045.1	GmMYB29 family protein	up-regulated at 2 hpi
916	0.11	0.47	0.59	0.00	XP_011011302.1	PREDICTED: AP2/ERF and B3 domain-containing transcription repressor RAV2-like	up-regulated at 2 hpi
1201	0.18	0.24	0.60	0.00	XP_011021732.1	PREDICTED: allene oxide cyclase 3, chloroplastic-like	up-regulated at 2 hpi
1250	0.51	0.00	0.65	0.00	XP_002312429.2	auxin efflux carrier family protein	up-regulated at 2 hpi
1448	0.15	0.34	0.61	0.00	XP_002317593.2	zinc finger family protein	up-regulated at 2 hpi
1583	0.06	0.70	0.50	0.00	XP_002300248.2	auxin-responsive GH3 family protein	up-regulated at 2 hpi
1889	-0.27	0.09	-0.29	0.06	XP_011028179.1	PREDICTED: transcription factor MYC2-like	up-regulated at 2 hpi
516	0.33	0.03	0.81	0.00	ABF81442.1	NBS-LRR type disease resistance protein	down-regulated at 1 dpi and/or 2 dpi
543	0.22	0.17	0.73	0.00	XP_002322142.1	enhanced disease susceptibility 1 family protein	down-regulated at 1 dpi and/or 2 dpi
560	0.42	0.01	0.83	0.00	XP_002297877.1	AP2 domain-containing transcription factor family protein	down-regulated at 1 dpi and/or 2 dpi
573	0.45	0.00	0.71	0.00	XP_006381476.1	Peroxidase 21 precursor family protein	down-regulated at 1 dpi and/or 2 dpi
651	0.20	0.20	0.78	0.00	XP_002323172.1	VQ motif-containing family protein	down-regulated at 1 dpi and/or 2 dpi
690	0.31	0.04	0.78	0.00	XP_002306976.2	catalase family protein	down-regulated at 1 dpi and/or 2 dpi
777	0.30	0.06	0.78	0.00	XP_002314935.1	bZIP family transcription factor family protein	down-regulated at 1 dpi and/or 2 dpi
938	0.16	0.32	0.61	0.00	XP_011017063.1	PREDICTED: 1-aminocyclopropane-1-carboxylate synthase 3-like isoform X1	down-regulated at 1 dpi and/or 2 dpi

1173	0.33	0.04	0.64	0.00	XP_011021969.1	PREDICTED: ethylene-responsive transcription factor ABR1-like isoform X1	down-regulated at 1 dpi and/or 2 dpi
1379	0.27	0.09	0.54	0.00	XP_006372897.1	putative glucosyltransferase family protein	down-regulated at 1 dpi and/or 2 dpi
1562	0.30	0.06	0.56	0.00	XP_002312060.1	ankyrin repeat family protein	down-regulated at 1 dpi and/or 2 dpi
119	0.39	0.01	0.92	0.00	ADW95385.1	Kunitz-type trypsin inhibitor	up-regulated at at multiple time points from 6 hpi to 4 dpi
537	0.43	0.00	0.71	0.00	XP_002312758.2	zinc finger family protein	up-regulated at at multiple time points from 6 hpi to 4 dpi
699	0.24	0.13	0.77	0.00	XP_006382787.1	calmodulin-like protein 6a	up-regulated at at multiple time points from 6 hpi to 4 dpi
755	0.36	0.02	0.73	0.00	XP_002301429.2	auxin-responsive family protein	up-regulated at at multiple time points from 6 hpi to 4 dpi
901	0.20	0.19	0.74	0.00	XP_002299891.2	zinc finger family protein	up-regulated at at multiple time points from 6 hpi to 4 dpi
1505	0.07	0.66	0.52	0.00	BAA94600.1	1-aminocyclopropane-1-carboxylate synthase	up-regulated at at multiple time points from 6 hpi to 4 dpi
1580	0.09	0.58	0.46	0.00	XP_011043122.1	PREDICTED: classical arabinogalactan protein 9-like	up-regulated at at multiple time points from 6 hpi to 4 dpi

Table S19. RT-qPCR primers used in this study.

Gene	Forward primer	Reverse primer
NBS-LRR (XP_006388824.1) No.1	AGTGGCGAAATGCAAGCAAG	AGTGAGTCGGCAGTCTTTGA
NBS-LRR (XP_006388824.1) No.5	AGACTGCCGACTCACTTGAA	GCACATAACGCAAGCCAACA
NBS-LRR (ABF81442.1)	ATTGCCTGCTGAGATTCGGA	TGCCTTGATGTGCCTCTACG
EDS1 (XP_002322142.1)	AGGTGACAGTTGCCCAAACA	ACTTTCTCACGCTCAACGCT
NDR1 (XP_002317572.1)	TCAGGGGCATAAAAAGAGTGCT	TCCACATTAGCCTCCACCCT
WRKY 72 (XP_011047157.1)	GACTTTGCCGAATCCAAGCC	CCGTCATTCATCGTTGGGGT
WRKY 47 (XP_002302808.1)	CCTAACGAGCAAGTCCCTGA	CACCGATAGTAGGCACGAGG
WRKY 65 (XP_002317397.1)	ATAGTGCTCAGGAAAGCGGTC	TTTCGCCCTTCAGCCTTGAG
PRs (XP_006379156.1)	TGTGGGTTGATGAGAAGGCT	TGCCGGGTGGATCATAGTTG
PRs (XP_002300292.2)	CGGGGGTCCTTATGGTGAAA	AGCACATCCTAGACGAACCG
PRs (XP_002306682.1)	AGGCAGGACTAAGGTATGGC	GTTGTAGCCTCCGCCAAGTT
Pathogenesis-related family protein (XP_002313936.2)	TTGTGGGTTGATGAGAAGGCT	GTTGCCGGGTGGATCATAGT
Jasmonic acid carboxyl methyltransferase family protein (XP_002307671.1)	GGCGTCCAACACCAGAACTT	ACCATAGAAAGAACCCGGCG
Lipoxygenase family protein (XP_006369133.1)	CTGGGCTTCGAGCATTGAGA	GTACGGCATCGTCTTGGGAA
Allene oxide synthase family protein (XP_002305405.1)	CGATGAAGCCAATCCCTGGT	CGGGAGGCATGTTGGTTTTG
Allene oxide cyclase 3, chloroplastic-like (XP_011021732.1)	ACACCTCAAACTCTCCAAAGCA	CCTCTGTCTCGCTCGTTGAT
Putative glucosyltransferase family protein (XP_002306205.2)	AGAGGCGGTAAACTGGTTGG	ACGGAGCCAGTTTCCTTTGA
Cytochrome P450 family (AHF20912.1)	AAACGTGGGTGCATAGGTGT	CCGGGATACACTTGTGGAGG
VQ motif-containing family protein (XP_002307385.1)	GGGCACCCACTACAGTTCTC	TGACCAGACCTCAATCCCGA

Jasmonic acid carboxyl methyltransferase family (XP_002307671.1)	CACGGACGAGAGTTGCTACC	TCGAGCCGATTGACACTGAA
Kunitz-type trypsin inhibitor (ADW95389.1)	ATCTTGCCAGTGTTCCGTGG	CACACCTTTCTTGTCGTCGG
Cytochrome P450 family (XP_002306491.1)	AGAGGATGGACGCTTTCTGG	TGTCCCTGCAACCAACATCA
18S ribosome RNA	CGAAGACGATCAGATACCGTCCTA	TTTCTCATAAGGTGCTGGCGGAGT
